# Integrative Modeling of Urinary Metabolomics and Metal Exposure Reveals Systemic Impacts of Electronic Waste in Exposed Populations

**DOI:** 10.3390/metabo15070456

**Published:** 2025-07-05

**Authors:** Fiona Hui, Zhiqiang Pang, Charles Viau, Gerd U. Balcke, Julius N. Fobil, Niladri Basu, Jianguo Xia

**Affiliations:** 1 Faculty of Agricultural and Environmental Sciences, McGill University, Ste-Anne-de-Bellevue, QC H9X 3V9, Canada; fiona.hui@mail.mcgill.ca (F.H.); charles.viau@mcgill.ca (C.V.); niladri.basu@mcgill.ca (N.B.); 2 Metacom Program Center, Leibniz Institute of Plant Biochemistry, 06120 Halle, Germany; gerd.balcke@ipb-halle.de; 3 School of Public Health, University of Ghana, Accra P.O. Box LG 13, Ghana; jfobil@ug.edu.gh; 4 West Africa Center for Global Environmental & Occupational Health, College of Health Sciences, Accra P.O. Box LG 13, Ghana; 5 Department of Microbiology and Immunology, McGill University, Montreal, QC H3A 2B4, Canada

**Keywords:** urine metabolomics, MS2, environmental health, heavy metals, electronic waste

## Abstract

**Background**: Informal electronic waste (e-waste) recycling practices release a complex mixture of pollutants, particularly heavy metals, into the environment. Chronic exposure to these contaminants has been linked to a range of health risks, but the molecular underpinnings remain poorly understood. In this study, we investigated the alterations in metabolic profiles due to e-waste exposure and linked these metabolites to systemic biological effects. **Methods**: We applied untargeted high-resolution metabolomics using dual-column LC-MS/MS and a multi-step analysis workflow combining MS1 feature detection, MS2 annotation, and chemical ontology classification, to characterize urinary metabolic alterations in 91 e-waste workers and 51 community controls associated with the Agbogbloshie site (Accra, Ghana). The impacts of heavy metal exposure in e-waste workers were assessed by establishing linear regression and four-parameter logistic (4PL) models between heavy metal levels and metabolite concentrations. **Results**: Significant metal-associated metabolomic changes were identified. Both linear and nonlinear models revealed distinct sets of exposure-responsive compounds, highlighting diverse biological responses. Ontology-informed annotation revealed systemic effects on lipid metabolism, oxidative stress pathways, and xenobiotic biotransformation. This study demonstrates how integrating chemical ontology and nonlinear modeling facilitates exposome interpretation in complex environments and provides a scalable template for environmental biomarker discovery. **Conclusions**: Integrating dose–response modeling and chemical ontology analysis enables robust interpretation of exposomics datasets when direct compound identification is limited. Our findings indicate that e-waste exposure induces systemic metabolic alterations that can underlie health risks and diseases.

## 1. Introduction

Electronic waste (e-waste) recycling has emerged as a significant environmental and public health concern due to the rapid global turnover of electronic devices [1,2]. Informal recycling practices, particularly in low-income countries, involve manual dismantling and open burning of discarded electronics, releasing toxic substances into the air, soil, and water. These activities expose workers and nearby communities to hazardous metals, including lead, cadmium, and chromium [3,4]. The lack of proper safety measures and environmental controls has led to increasing evidence of biological harm, prompting urgent attention toward monitoring, regulation, and safer, sustainable recycling solutions to mitigate long-term health risks [5,6,7,8,9,10,11,12].

Liquid chromatography coupled with mass spectrometry (LC-MS) has become a crucial tool for investigating environmental exposures and associated metabolic responses. LC-MS-based untargeted metabolomics can provide comprehensive snapshots of metabolic profiles in biological samples [13,14,15,16,17]. A major challenge lies in the accurate identification of compounds from tens of thousands of features detected during analysis. To help improve compound annotation, a common practice is to further characterize MS1 features using associated MS2 spectra. MS1 signals are used to quantify compounds and provide information on their molecular weight, while MS2 spectra offer additional fragmentation patterns that are essential for structural elucidation. Compound identification is usually performed by comparing experimental spectra against reference spectra from databases.

A considerable gap remains between the sheer number of MS1 features detected and the portion of those features that can be identified using information from MS2 spectra [18,19]. There is a significant lack of reference standards for many compounds, especially for novel or less-studied chemicals [20,21]. The issue becomes more prominent in exposomics studies, where environmental and exogenous compounds are often present at trace levels and fall outside the scope of well-characterized metabolite libraries [22]. The inability to match MS2 spectra with known compounds results in many features being classified as “unknown” or “putative”, offering little clarity about their biological or environmental relevance. This large gap between detected features and compounds identified underscores the need for new strategies to improve confidence in metabolite annotation.

One promising strategy involves the use of ontology-based classification systems, which can provide insights even when compound identities cannot be precisely determined. Chemo-ontologies categorize compounds based on structural or functional properties, offering a way to group detected metabolites into broader chemical classes [23,24]. While the exact identity of a compound may remain elusive, structural information can often be gleaned by analyzing MS2 fragmentation patterns. These patterns may suggest the chemical class of a compound with greater confidence, thus allowing researchers to infer its potential biological roles. For instance, the fragmentation patterns can point to whether a compound belongs to broad classes such as xenobiotics, lipids, or microbial metabolites, even if the exact molecular formula remains undetermined [25,26].

Importantly, identifying which chemical classes are perturbed in a biological sample can provide meaningful insights into possible exposures and associated mechanisms of action. For example, enrichment of certain xenobiotic or environmental pollutant classes in exposed populations, such as e-waste workers, can suggest exposure to hazardous chemicals, while the depletion of key metabolic classes may signal disruptions in metabolic pathways. This approach has been proven useful in prior studies in the context of exposures to air pollution and e-waste, where alterations in lipid classes (such as fatty acids and sphingolipids) and amino acid (such as methionine and tryptophan) metabolism were identified and served as a bridge between high-resolution mass spectrometry data and biological interpretation [27,28,29,30]. By focusing on chemical class-level analysis, researchers can make meaningful biological interpretations even in the absence of a complete compound identification. Complementing conventional database-search-based approaches with ontology-based classification enables improved utilization of the vast amount of MS1 and MS2 data, ultimately enhancing our understanding of the exposome and its impacts on health.

In this study, we address the aforementioned challenges by implementing a workflow that integrates conventional MS2-based compound annotation with ontology classification, providing a more structured and interpretable view of the exposome in a highly exposed population. Specifically, we focus on urine samples from e-waste workers from the Agbogbloshie site in Ghana as detailed previously [31,32,33]. By leveraging extensive reference libraries and focusing on chemical class enrichment, we aim to bridge the gap between feature detection and biological insight, offering a comprehensive analysis of the biological impacts of e-waste exposure.

## 2. Materials and Methods

### 2.1. Sample Selection

Urine samples of 91 male e-waste workers from Agbogbloshie (Ghana) and 51 non-e-waste worker controls with comparable age and cultural profiles from Madina Zongo (Ghana) were included in this study. These samples were from the GEOHealth II study (from March 2017 to October 2018) as detailed previously [31,32,33]. The demographic and lifestyle metadata of the participants, including age, BMI, smoking status, stress, and years of work, were summarized and described in our previous study [15]. Informed consent was obtained from all study participants, and ethics approvals were obtained from the Ethical and Protocol Review Committee at the College of Health Sciences, University of Ghana (protocol identification number CHS-ET/M.4-P 3.9/2015–2016) and the Research Ethics Office of McGill University (Study ID: A05-M26-16B).

### 2.2. Metal Quantification

A total of twenty-two metals were measured in urine samples: silver (Ag), arsenic (As), barium (Ba), calcium (Ca), cadmium (Cd), cerium (Ce), chromium (Cr), copper (Cu), europium (Eu), iron (Fe), lanthanum (La), magnesium (Mg), neodymium (Nd), nickel (Ni), lead (Pb), rubidium (Rb), selenium (Se), strontium (Sr), terbium (Tb), thallium (Tl), yttrium (Y), and zinc (Zn). Following nitric acid digestion, urinary metal concentrations were measured by inductively coupled plasma mass spectrometry (ICP-MS; Varian 820MS, SpectraLab Scientific Inc., Markham, ON, Canada), using external calibration curves with element-specific standards. A detailed quantification method can be found in a previous study by Takyi et al. [33].

### 2.3. LC-MS/MS Analysis and Data Processing

Urine samples underwent liquid chromatographic separation followed by MS1 and MS2 acquisition using Thermo Scientific™ UltiMate™ 3000 ultra-high-performance liquid chromatography (UHPLC) coupled with Q-Exactive™ Orbitrap high-resolution mass spectrometry (HRMS) system (Waltham, MA, USA). Experimental procedures were as detailed previously [15,34]. In brief, a dual-column and dual-ionization approach was opted to ensure broad coverage for metabolomics profiling, using both hydrophilic interaction liquid chromatography (HILIC) and reversed-phase C18 columns, with data acquired under both positive and negative electrospray ionization (ESI) modes.

Raw data were converted to mzML format using msconvert from ProteoWizard (v3.0.23156) [35]. MS1 spectra were processed using the asari algorithm (v1.13.1) with default parameters [36], based on its high sensitivity in trace-level features detection as shown in our previous exposomics study [15]. MS2 spectra were processed and matched against spectra databases for compound identification using MetaboAnalystR 4.0 [37]. Furthermore, SIRIUS suite (v6.0.0) [26,38] was employed in parallel to infer chemical class and ontology, also with default settings. The predicted ClassyFire ontology levels, including superclass, class, and subclass annotations, were used to group compounds. A confidence threshold of ≥80 (on a scale of 0–100) or 0.8 (on a 0–1 scale) was applied to spectral matching-based compound identification and predicted chemical classification, respectively, to retain reliable annotations. Identified compounds were further categorized based on exposure origins, functions, and toxicological properties using curated databases and literature references.

### 2.4. Bioinformatics, Statistical Analyses and Modelling

To explore relationships between urinary metal concentrations and metabolomic features, both correlation and regression-based methods were applied. Pearson and Spearman correlation matrices were first constructed to assess linear and monotonic associations, respectively, between individual metal concentrations and metabolite intensities. Subsequently, multiple linear regression models were fitted for each metal-metabolite pair, adjusting for relevant covariates including age, sex, body mass index (BMI), smoking status, and self-reported stress. To capture nonlinear exposure–response patterns, four-parameter logistic (4PL) regression curves were also applied to the same pairs, allowing detection of threshold effects, saturation dynamics, and sigmoidal dose–response behaviour. All *p*-values were corrected for multiple comparisons using the Benjamini–Hochberg procedure at a false discovery rate of 5%; adjusted *p*-values < 0.05 were considered significant. Lastly, metabolite features identified through linear and 4PL models were compared to characterize the complexity and diversity of biological responses to metal exposure. The above approach enabled the detection of distinct profiles that may reflect physiological thresholds or adaptive processes. All statistical analyses and visualizations were performed in R, using the protti package [39] for 4PL modelling, plotly [40] for interactive data visualization, and other base functions and standard libraries for data manipulation and plotting. Functional and pathway analysis were performed using MetaboAnalyst 6.0 [41] and MetaboAnalystR 4.0 [34].

## 3. Results

### 3.1. Overview of MS1 and MS2 Raw Data Processing

**Figure 1:**
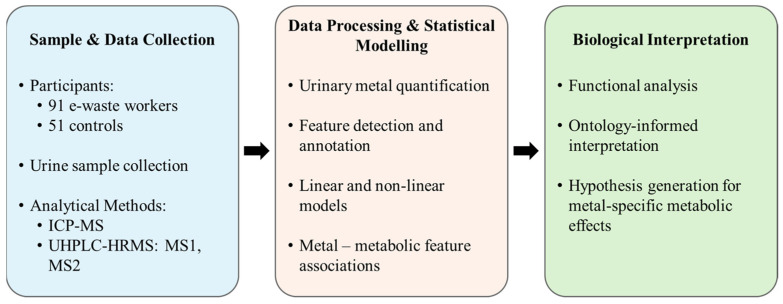
Overview of the data processing and analysis pipeline.

MS1 data analyzed through asari yielded a substantial number of features across both HILIC and C18 columns under negative and positive ionization modes. A graphical summary of the analytical methods and metabolomics data analysis pipeline is presented in Figure 1, and a detailed breakdown of feature counts per sample and classification levels is provided in Supplementary Table S1. In brief, in urine samples from e-waste workers, around 50,000 features were detected in each ionization mode of the HILIC column, while the C18 column yielded more than double the number of features. Comparatively, urine samples from the control group exhibited fewer detected features, at approximately 70% across both modes and columns. Subsequent MS2 spectral matching against spectra libraries allowed compound identification for >700 high-confidence features in e-waste worker samples and >500 features in the control group. These results consistently show a higher number of detected features in e-waste worker samples, suggesting broader and more complex chemical exposure profiles than in the control population group.

### 3.2. Metal Quantification in Urine Samples

**Figure 2:**
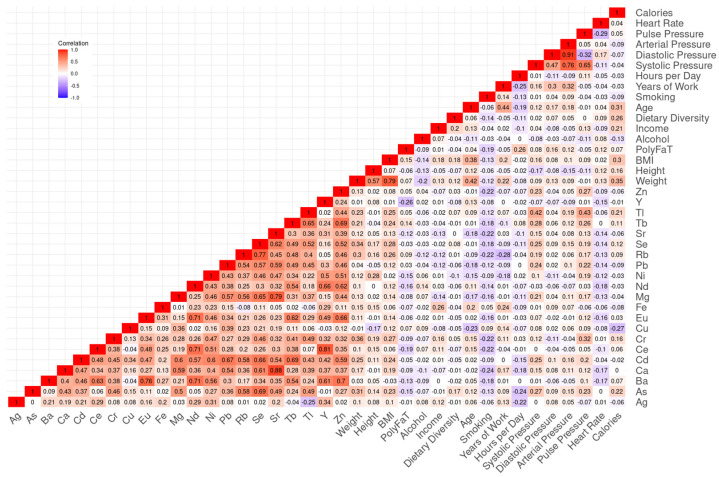
Correlation heatmap among urinary metal levels and participant characteristics.

Analysis of urinary metal concentrations revealed complex patterns of co-exposure and biological interaction. Several metals exhibited strong positive correlations (Spearman’s rank correlation ρ > 0.7), particularly among elements belonging to the same chemical group (Figure 2). For instance, a strong network of correlations was observed among rare earth elements, including neodymium (Nd), cerium (Ce), europium (Eu), and yttrium (Y), as well as with barium (Ba). These patterns likely reflect the co-release of metals during dismantling and thermal processing of components such as circuit boards, conductors, and electrodes in informal e-waste recycling sites. Notably, the three alkaline earth metals—strontium (Sr), calcium (Ca), and magnesium (Mg)—were also highly correlated. Biological pathways in mineral regulation and transport. While Ca and Mg are essential minerals involved in numerous physiological processes, Sr biologically mimics calcium and may act as its surrogate; their correlation patterns likely reflect a combination of shared biological pathways in mineral regulation and transport, and co-exposure from environmental sources.

When examining associations between urinary metal levels and participant characteristics, expected physiological correlations were observed, for instance, between weight and BMI, or amongst arterial, diastolic, and systolic blood pressure. Interestingly, we also identified weak positive correlations between multiple urinary metals and elevated blood pressure, suggesting a potential link between metal exposure and health risk. Comparison of urinary metal concentrations between the e-waste worker group and the control group revealed comparable levels across many elements, despite the occupational nature of exposure in one group. This supports the hypothesis of environmental spillover, where contaminants released in high-exposure areas disperse into surrounding regions or allied exposure sources that generate similar agents, leading to similar internal metal burdens even in populations not directly involved in recycling activities.

### 3.3. Categorical- and Ontology-Based Classification

**Figure 3:**
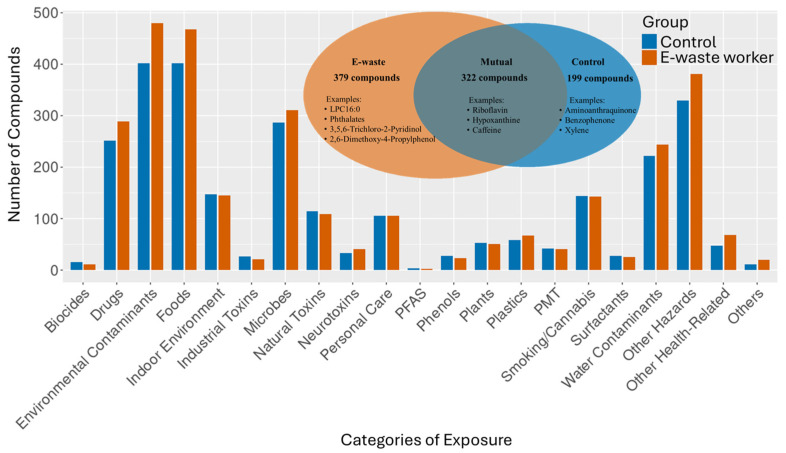
Metabolite compounds categorical profiles. The Venn diagram shows a comparison of MS2-annotated compounds detected in the e-waste worker and control groups. PFAS—per- and polyfluoroalkyl substances; PMT—persistent, mobile, and toxic substances.

A comparison of compound identities between the e-waste workers and the control group revealed that shared features are largely core metabolic compounds or ubiquitous environmental compounds (Figure 3). For instance, riboflavin (vitamin B2) and hypoxanthine are essential metabolites involved in energy production and cellular function, whereas caffeine is a common natural chemical from dietary sources. In contrast, compounds unique to e-waste workers included LPC16:0, a lysophospholipid linked to oxidative stress, as well as synthetic and industrial chemicals like 2,6-dimethoxy-4-propylphenol (a flame retardant), 3,5,6-trichloro-2-pyridinol (an organochlorine), and various phthalates, all of which are frequently present in e-waste plastics. Compounds found exclusively in the control group reflected lifestyle or environmental exposures typical of non-occupational sources, including xylene, benzophenone, and aminoanthraquinone, which are associated with pollutants, cosmetic products, and pharmaceuticals, respectively. Notably, distinct patterns in the kynurenine pathway were observed between the two groups. The e-waste worker group showed elevated levels of 3-hydroxyanthranilic acid and N-methylanthranilic acid, while the control group exhibited higher levels of anthranilate and formylanthranilic acid. This metabolic shift suggests a redirection of tryptophan metabolism upon exposure to toxins and pollutants from e-waste.

To gain holistic insight beyond individual compound identification, we grouped the identified metabolites into various categories. Despite the subtle differences in the number of metabolite compounds identified, we observed that the metabolite profiles of the e-waste group and control group were comparable in terms of the types of categories represented. A great number of metabolites fell predominantly into categories such as environmental contaminants, hazardous substances, food-derived compounds, microbial metabolites, and pharmaceutical/drug-related compounds.

In addition, we performed chemo-ontology-based classification using *SIRIUS* fragmentation-informed predictions. Although the e-waste worker group displayed a higher total number of annotated compounds (Supplementary Table S1), the relative distribution across chemical ontology categories was notably similar to that observed in the control group (Supplementary Figure S1). An important exception was the marked enrichment of organic nitro compounds, especially nitrobenzenes, in the e-worker samples. Because nitrobenzenes arise from the thermal dismantling and combustion of plastics and circuit boards, their presence represents a distinct occupational signature superimposed on the shared regional background and points to additional risks of oxidative and xenobiotic stress in e-waste recyclers. This finding suggests that while occupational exposure contributes additional compound diversity, there is also significant overlap in exposure profiles, likely due to regional dispersion of e-waste-related pollutants into the surrounding environment.

### 3.4. Linear Relationship Between Urinary Metals and Metabolites

To assess possible molecular changes associated with metal exposure, we investigated associations between urinary metal concentrations and metabolite intensities using both Pearson and Spearman correlation analyses, capturing linear and monotonic trends, respectively. Significant correlations identified through these methods were subsequently evaluated using multivariable linear regression models, adjusted for potential confounders including age, sex, BMI, smoking status, stress, and occupational duration. Most associations observed in the correlation analyses remained robust after adjustment, supporting the consistency of these exposure-metabolite relationships. Several metals exhibited a high degree of connectivity with urinary metabolite features. Notably, arsenic (As), calcium (Ca), magnesium (Mg), lead (Pb), rubidium (Rb), selenium (Se), and strontium (Sr) demonstrated widespread linear associations, identified consistently in both Pearson and Spearman correlation matrices (Table 1). These metals correlated with 100s to 10,000s of metabolite features, underscoring their broad biochemical influence. In contrast, cadmium (Cd), copper (Cu), and thallium (Tl) showed stronger associations in Spearman analyses, but weaker or absent signals in Pearson correlation, indicating nonlinear or saturation-type effects not well captured by linear models. In particular, Cd and Cu were associated with a large number of metabolites detected specifically in the C18 column under positive ionization mode, suggesting a preferential impact on hydrophobic or semi-polar metabolic pathways.

In total, over 120,000 statistically significant linear associations were identified across all metals and metabolite features. While only about 1% of these features had direct compound annotations, incorporating MS2-based chemical class predictions increased the proportion of interpretable associations to 3–5%. Among the chemically annotated features, urinary metal levels were frequently associated with compounds classified as carboxylic acids and derivatives, allyl-type 1,3-dipolar compounds, purine nucleotides, benzenes and substituted derivatives, organooxygen, and organonitrogen compounds.

Despite the large number of associations, the majority of effect sizes were modest, with linear regression coefficients typically ranging between 0.5 and 1.0. This pattern suggests subtle but widespread metabolic shifts, particularly in chemical classes such as fatty acyls, steroids, and carboxylic acids. These trends may reflect low-level but persistent disruptions in lipid metabolism, hormonal balance, and organic acid homeostasis, without large-scale perturbations of individual pathways. Linear associations between annotated metabolites and each metal are provided in Supplementary Figure S2.

### 3.5. Nonlinear Relationship Between Urinary Metals and Metabolites

To capture complex, non-monotonic, or threshold-dependent biological responses to metal exposure, we employed four-parameter logistic (4PL) regression modeling across all metal-metabolite pairs to reveal nonlinear associations that were not evident through standard linear regression or correlation analysis. A total of twelve urinary metals, including As, Ca, Cd, Cr, Cu, Mg, Pb, Rb, Se, Sr, Tl, and Zn, showed significant associations with metabolite features when modeled using the 4PL approach. Among these, Cr and Zn were notable in that their metabolite associations were uniquely captured by the 4PL model, indicating the presence of nonlinear or threshold-like biological responses that would have been missed by linear models. Nonlinear associations between annotated metabolites and each metal are provided in Supplementary Figure S3.

**Figure 4:**
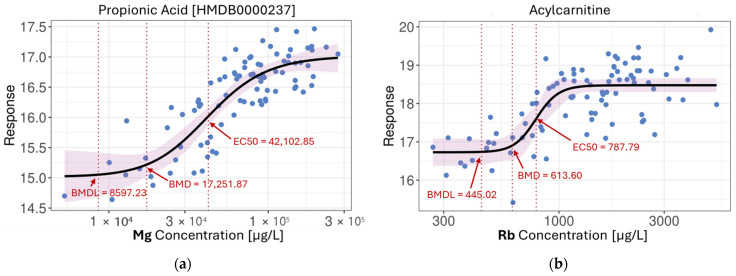

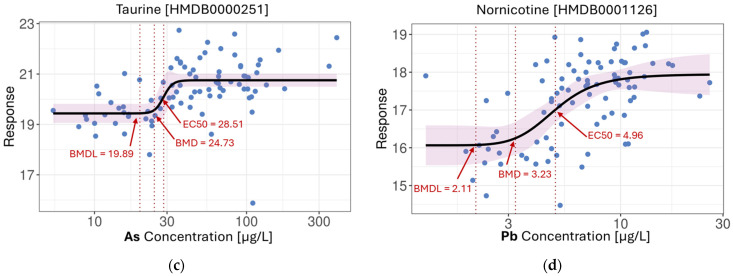
4PL nonlinear models: (**a**) Propionic acid in response to increasing levels of Mg; (**b**) Unidentified compound from the acylcarnitine group in response to increasing levels of Rb; (**c**) Taurine in response to increasing levels of As; (**d**) Nornicotine in response to increasing levels of Pb. BMD—benchmark dose; BMDL—benchmark dose lower confidence limit; EC50—half maximal effective concentration. (BMD and BMDL were reported at 10% of the full response range. Arrows and labels were added post hoc to indicate key values).

Among Spearman-correlated pairs reported in the previous section, 66.9% (*n* = 82,833) were well-fitted by 4PL curves, confirming that many monotonic relationships were in fact nonlinear in nature. Furthermore, 50.4% (*n* = 36,032) of the linearly associated features also fit robust 4PL curves, emphasizing the added resolution provided by this model in detecting sigmoidal dynamics. Overall, across all metals, we identified over 360,000 significant nonlinear associations, substantially expanding the landscape of metal-related metabolic effects. A few examples of 4PL metal-metabolite models are illustrated in Figure 4. However, despite the large number of associations, only around 0.5% of features could be directly identified at the compound level, while an additional 2% were classifiable via MS2-informed chemo-ontologies. As shown in Figure 4b, one such feature exhibited a clear dose–response relationship with Rb. While its exact identity remains unknown, chemo-ontology annotation suggests it likely belongs to the acylcarnitine class, highlighting the utility of structural classification in interpreting untargeted metabolomics data.

The metabolite classes most responsive to metal exposure included carboxylic acids, organooxygen compounds, imidazopyrimidines, benzenes, fatty acyls, and steroids. In particular, Cd displayed strong nonlinear associations with numerous compounds in the carboxylic acid, fatty acyl, and organooxygen categories, often exhibiting low EC50 values, suggesting that these classes are highly sensitive to even minimal cadmium exposure. These findings likely reflect early metabolic responses involving lipid oxidation, organic acid dysregulation, and oxidative stress pathways.

The 4PL model also revealed distinct response shapes. For instance, both Pb-nornicotine and Mg-propionic acid associations exhibited classic sigmoidal curves, while the As-taurine relationship displayed a threshold-like behaviour, where metabolite levels remained stable at low metal concentrations before rising sharply and plateauing at higher exposures. The benchmark dose (BMD) and EC50 values were also closely aligned, suggesting steep dose–response curves with minimal early response and a relatively narrow dynamic range. These patterns may reflect underlying homeostatic buffering, adaptive stress responses, or toxicodynamic saturation, all of which are biologically relevant yet challenging to detect with conventional linear modeling.

### 3.6. Comparison of Model-Specific Metal-Metabolite Associations

To further interpret the biological significance of metal-metabolite relationships, we compared features that were exclusively identified through linear regression versus those uniquely captured by the 4PL model. This comparison revealed distinct profiles of metabolite responses, suggestive of different underlying biological mechanisms. Metabolites showing significant associations only in linear models tended to exhibit proportional, monotonic relationships with increasing metal concentrations. These patterns are consistent with steady-state physiological responses, in which metabolite levels incrementally reflect changes in exposure. Many of these features were identified as endogenous compounds, including cytosine, niacinamide (vitamin B3), and L-valine, all of which are integral to nucleotide synthesis, redox balance, and amino acid metabolism, respectively. Their linear behaviour likely reflects gradual metabolic adaptation or regulation under subtoxic exposure conditions. In contrast, metabolites uniquely detected through 4PL models exhibited nonlinear, often sigmoidal dose–response curves, pointing to more complex and threshold-based biological regulation. These relationships suggest the presence of buffering systems, saturation kinetics, or adaptive defense mechanisms that only activate beyond a certain exposure threshold. Annotated compounds in this group included oxazepam (a benzodiazepine metabolite), nornicotine (a nicotine derivative), taurine (an osmolyte and antioxidant), and chloramphenicol (an antibiotic). Their nonlinear response profiles may reflect pharmacological sensitivity, detoxification dynamics, or xenobiotic processing thresholds.

Together, these model-specific associations underscore the biological plausibility of response types: while linear associations reflect ongoing homeostatic regulation, nonlinear patterns signal disruption, activation, or overload of biological systems in response to environmental metal exposures. The integration of both modeling strategies thus provides a more nuanced understanding of exposure biology, highlighting the value of complementary statistical approaches in exposomic research.

### 3.7. Functional and Biological Pathway Analysis

**Figure 5:**
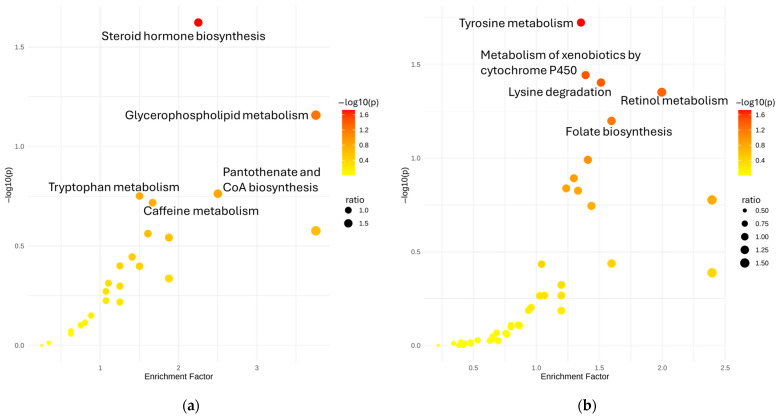
Disrupted pathways on (**a**) HILIC-negative mode and (**b**) HILIC-positive mode.

To gain biological insight into the metabolic alterations associated with metal exposure, we conducted pathway enrichment analysis using annotated and classified metabolite features. The resulting enrichment plots (Figure 5) highlight several biologically relevant pathways that were significantly overrepresented among metal-associated metabolites.

In HILIC-negative mode, pathways such as steroid hormone biosynthesis, glycerophospholipid metabolism, and pantothenate and CoA biosynthesis were significantly enriched. These findings align with the results from both linear and nonlinear modeling, where compounds related to lipids, steroids, and fatty acyls were frequently associated with metals such as Cd, Cu, and Pb. The enrichment of tryptophan metabolism further supports observations of altered kynurenine pathway intermediates in e-waste workers, suggesting oxidative stress and immune imbalance as part of the biological response to exposure. In HILIC-positive mode, the most enriched pathways included tyrosine metabolism, retinol metabolism, lysine degradation, and metabolism of xenobiotics by cytochrome P450. These enrichments suggest disruption of amino acid metabolism, vitamin A-related functions, and activation of detoxification mechanisms, likely reflective of systemic attempts to buffer or metabolize accumulated toxicants. The involvement of cytochrome P450 metabolism corresponds well with nonlinear associations involving compounds like oxazepam, taurine, and chloramphenicol, which are known substrates of xenobiotic-metabolizing enzymes and showed sigmoidal dose–response relationships with metals like Pb and As.

Taken together, these results reinforce earlier findings that metals influence lipid regulation, amino acid turnover, antioxidant defense, and detoxification pathways in e-waste-exposed individuals. The complementary enrichment patterns observed in both ionization modes demonstrate the broad metabolic footprint of metal exposure and highlight the value of integrating chromatographic diversity with functional annotation in exposomics studies.

## 4. Discussion

In this study, we presented a comprehensive exposomic investigation into the biological impacts of chronic metal exposure among e-waste workers and nearby control residents, using untargeted urinary metabolomics and a multi-model statistical framework. By integrating MS2-informed chemo-ontology with both linear and nonlinear regression modeling, we uncovered a diverse array of metabolic perturbations associated with urinary metal levels, offering new insights into the systemic effects of e-waste exposure. Our results confirmed that e-waste workers exhibited a broader and more chemically diverse metabolomic profile, with increased detection of both endogenous and exogenous compounds, as well as higher rates of MS2 annotation and chemical classification. Notably, several exogenous compounds uniquely enriched in the e-waste group, such as flame retardants, phthalates, and nitrobenzenes, point to occupational exposure to synthetic and industrial pollutants commonly released during informal recycling processes. These compounds are known to act as endocrine-disrupting chemicals, toxicants, and oxidative stress inducers [42,43,44,45]. Their detection in urine indicates active exposure and systemic absorption. Importantly, exposures to these pollutants were mirrored by alterations in endogenous metabolites observed uniquely in e-waste workers’ samples, which suggested a shift toward pro-inflammatory and oxidative stress-related pathways, potentially driven by toxicant exposure and immune dysregulation [46,47,48]. However, although the e-waste group has a higher number of metabolites in each compound category, samples from the control group exhibited a similar trend. The similar chemical ontology distributions observed in the community controls, along with measurable levels of all heavy metals investigated, including the rare earth elements, which are not typically found in general urban or industrial pollution and are more commonly associated with specialized sources such as electronics, suggest potential environmental spillover. This reinforces the concern that hazardous contaminants extend beyond the boundaries of e-waste processing zones. Although the participants from nearby community did not directly work with e-waste recycling and were located some distance away from the site, the contamination from e-waste may well spread through air, water, soil, etc., raising the issue that inappropriate treatment of e-waste may have much wider effects, not only impacting the workers but also populations in the greater region [49,50,51,52]. Alternatively, there may also be other sources of exposures in a large urban center like Accra, such as vehicular exhaust fumes or emissions that release similar chemical agents into the ambient environment, thus raising the importance of source apportionment and risk assessment studies [53,54,55].

Urinary metal quantification revealed strong co-occurrence patterns among chemically related elements, such as Sr-Ca-Mg (alkaline earth metals) and Nd-Ce-Eu-Y (rare earth elements). In the case of rare earth elements, these metals are not considered essential and are not known to participate directly in human biological or metabolic pathways. Therefore, their levels in urine are likely to reflect environmental exposure, particularly from co-release during informal e-waste processing. In contrast, Ca and Mg are essential minerals involved in numerous biological functions, including bone health, nerve and muscle function. Sr, while not essential, closely mimics calcium in biological systems and can act as a surrogate in absorption and tissue incorporation. The interplay among Ca, Mg, and Sr has been well-documented [56,57,58], and their strong correlation in our data may reflect shared biological handling mechanisms—such as absorption, cellular transport, and regulatory pathways—in addition to common environmental sources. Thus, both external exposure and internal physiological regulation likely contribute to the observed patterns in this case. A previous study using blood samples uncovered numerous metabolites correlating with various metals (Se, Tb, Cu, Mg, Ca, Sr, Rb, Tl, Zn, Mn, Cd, Fe, La, Ce, Eu, Nd, Pb, and Y), half of these metals can also be detected in urine and corroborate a similar correlation with metabolite levels [15]. Our use of urinary concentrations provides complementary insight due to their renal excretion. While blood metals tend to capture biologically active exposure levels influencing metabolite concentrations more directly, urinary metals reflect a downstream or detoxification phase of exposure, which can cause attenuation or delay in observed metabolic responses. These findings in urine samples complement prior blood-based studies by offering a downstream view of exposure metabolism and suggest that both matrices provide valuable, yet distinct, biological insights. Furthermore, the detection of weak but positive correlations between certain metals and elevated blood pressure supported previous literature linking heavy metal exposure to increased cardiovascular risk, reinforcing the public health relevance of these exposures [59,60].

The statistical modeling framework applied in this study allowed us to discern not only the prevalence but also the complexity of metal-metabolite relationships. Linear regression modeling revealed over 120,000 associations, particularly with As, Ca, Mg, Pb, Rb, Se, and Sr. Most of these associations involved small effect sizes and pointed toward subtle alterations in lipid metabolism, amino acid turnover, and organic acid regulation. Metals such as Cd and Cu displayed a preference for associations with hydrophobic metabolites detected in the C18 column positive mode, hinting at chemical-specific interaction profiles. Beyond linearity, our use of the four-parameter logistic (4PL) model revealed over 360,000 nonlinear associations, many of which were missed by traditional correlation methods. Cr and Zn, in particular, were captured almost exclusively through nonlinear fits, emphasizing the added value of flexible modeling approaches. We selected the 4PL model with the underlying assumption that increasing metal concentrations could alter the abundances of certain metabolites in a dose-dependent manner [61,62]. The 4PL model is frequently used in pharmacokinetic and toxicological studies to fit a sigmoidal curve with four parameters—the bottom and top asymptotes, the EC50, and the slope—which provides the advantage of better biological interpretability [63,64]. However, while 4PL models are well-suited for capturing canonical dose–response behaviour and provide interpretable EC50 estimates, they do impose a specific sigmoidal shape and may fail to capture more complex relationships. Moreover, since our current modeling approach evaluates each metal-metabolite pair independently, it cannot resolve whether observed associations are driven by shared biological pathways or confounded due to correlated co-exposures. These limitations highlight the need for future studies to include more flexible, multivariate mixture modeling approaches [65,66], potentially complemented by experimental validation, to identify specific causal relationships and disentangle the joint effects of co-occurring exposures. The detection of dose–response curves with threshold effects (e.g., As-taurine) or sigmoidal dynamics (e.g., Pb-nornicotine) underscores the biological plausibility of metabolic buffering, saturation, or toxicity thresholds being activated by environmental exposures. These nonlinear relationships highlight processes such as oxidative stress responses, detoxification, and xenobiotic overload that cannot be accurately captured through linear models alone. These findings are consistent with the literature findings where increased amount of taurine has been shown to have a protective role against arsenic-induced damage [67,68,69,70]. While in the case of Pb-nornicotine, although initial intuition may attribute the finding towards lifestyle-based co-exposure, namely cigarette use [71], previous studies have shown that smoking alone did not strongly associate with Pb, whereas individuals who smoke as well as working in environments with heavy metal exposure showed a dose–response relationship between smoking and Pb levels [72]. This may suggest an alternative explanation that the Pb-nornicotine association is due to Pb interfering with nicotine metabolism or clearance, potentially through disruption of liver enzyme activity or detoxification pathways. The dose–response behavior suggests that beyond a certain threshold, Pb exposure may induce adaptive or toxicological changes in xenobiotic metabolism, resulting in elevated systemic levels of nornicotine [73,74]. In either case, further investigation and validation are warranted to confirm the exact mechanism of action. These findings demonstrate how preliminary results from untargeted metabolomics studies may help generate reliable mechanistic hypotheses from a vast number of candidates for ensuing experimentation and validation.

The contrast between linear-only and nonlinear-only associations reflects fundamental differences in biological response types. While linear patterns may represent steady, homeostatic regulation of metabolic pathways under low or moderate exposure, nonlinear responses likely mark points of metabolic transition or dysregulation. The implicated compounds, ranging from core metabolites like niacinamide and valine to stress-responsive agents like oxazepam and taurine, reflect a continuum of biochemical reactivity shaped by the intensity and duration of metal exposure. These results underscore the potential for passive, non-occupational exposure in nearby populations and reinforce the value of using chemical ontologies to assess systemic exposure patterns when full compound identification is limited. Collectively, these findings validate a dual-model statistical approach for exposomic data interpretation and underscore the value of chemical ontology in resolving otherwise unidentifiable metabolomic signals. They also confirm and extend previous reports linking metal exposure to disruptions in lipid profiles, amino acid metabolism, and redox balance in occupational and environmental settings [75,76]. Importantly, this work illustrates how regional populations not directly involved in recycling may still exhibit metabolomic signatures of contamination, reinforcing the need for broader environmental monitoring and health risk assessment.

From a methodological standpoint, our approach offers practical utility for longitudinal or multi-batch studies where technical variability limits direct MS1 intensity comparisons. This may be particularly relevant for toxic exposure types of studies, where many studies are carried out retrospectively, challenges such as cross-sectional study design and limited demographic diversity may be common and pose constraints on generalizability and causal inference. Informal e-waste recycling sites often involve sustained hazardous exposures, offering a unique opportunity to investigate exposure-related health and biological responses. However, the uncontrolled and informal nature of these environments also makes it difficult to implement long-term, rigorously matched and coordinated study designs [77,78]. To mitigate this, we propose using MS2-informed interpretation as a bridging strategy, enabling indirect, qualitative, class-level comparisons. This allows us to identify recurring patterns of metabolic response across independent studies and cohorts without requiring direct MS1-level normalization. It is important to emphasize that associations identified through this approach are hypothesis-generating, intended to guide more targeted and mechanistically focused studies in broader and more controlled populations for downstream validation. Looking forward, expanding spectral libraries, improving MS2 acquisition strategies, and integrating multi-omics layers (e.g., proteomics, transcriptomics) will be crucial to help understand biological responses to environmental exposures and health outcomes.

A key limitation of this study, which is often a limitation of untargeted metabolomics in general, particularly in complex exposomic studies, lies in the substantial proportion of MS1 features that remain unidentifiable. This challenge stems from both instrumental constraints and workflow-level limitations in current acquisition strategies. While Orbitrap MS platforms offer excellent MS1 sensitivity and mass accuracy, they operate with relatively slow MS2 acquisition speeds, limiting the number of tandem mass spectra that can be collected per run. In addition, our use of data-dependent acquisition (DDA), chosen for its ability to generate high-quality MS2 spectra, comes with the trade-off of prioritizing the most abundant ions for fragmentation, often excluding low-abundance or transient features [79,80]. These factors result in incomplete MS2 spectral coverage, especially for features that may be biologically or toxicologically informative but are poorly ionizing or present at low concentrations. In addition, spectral matching using MS2 data remains constrained by the incompleteness of public reference libraries and fragmentation variability across platforms and conditions. Matching against large, general-purpose databases also introduces additional ambiguity, further limiting confidence in structure-level annotation [81,82]. Although tools like chemical ontology prediction and MS2-based classifiers enhance interpretability by assigning chemical classifications to unidentified features, the overall annotation rate after filtering by confidence score still constrains biological inference. This bottleneck underscores the need for improved LC-MS technologies and the continued expansion of high-quality spectral libraries, not only used for spectral matching-based compound identification, but also context-dependent chemical ontology inference based on fragmentation pattern and similarity to known spectra.

Future efforts in these areas will be essential to increase annotation coverage, enable more comprehensive biological interpretation, and support target validation in environmental health research. From an exposomics perspective, this study represents an initial step toward characterizing metal-associated metabolomic responses in an e-waste-exposed population. While informative, it provides a partial view of the exposome and does not account for the potential influence of co-pollutants commonly present in e-waste environments, such as plasticizers, flame retardants, or other organic toxicants. As a result, the analysis may not fully capture interactive or additive effects of complex exposure mixtures. In addition, the current analysis focuses primarily on modeling metal exposures in relation to metabolic changes, but it may also be of great interest to examine how certain phenotypes or metabolic features may predict or correlate with multiple exposures, a gap that could be addressed through inverse, exposome-wide association studies. Future work should expand the analytical framework to incorporate additional e-waste-related pollutants and apply multi-pollutant modeling approaches to better understand interactive, synergistic, or cumulative effects on the metabolome [83,84].

## 5. Conclusions

This study demonstrates the power of integrating untargeted urinary metabolomics with chemical ontology and dose–response modeling to characterize the biological impacts of e-waste-related metal exposure. By applying both linear and nonlinear (4PL) frameworks, we uncovered a wide spectrum of metal-metabolite relationships, ranging from steady-state physiological responses to threshold-dependent metabolic disruptions. The detection of diverse chemical classes highlights the systemic effects of chronic metal exposure on metabolic health. Our findings reveal that informal e-waste recycling not only affects workers directly involved but also has implications for surrounding communities through environmental spillover. Together, these results advance our understanding of environmental toxicology at the molecular level and support the development of exposure biomarkers that can inform risk assessment and public health interventions.

## Figures and Tables

**Table 1:** Significant linear associations between MS1 features and various urinary metals by Pearson and Spearman’s rank correlations. MS1 features detected in HILIC and C18 columns, both negative (ESI^−^) and positive ionization (ESI^+^) modes.

Urinary Metal	Association	Pearson Correlation				Spearman’s Rank Correlation			
		HILIC Column		C18 Column		HILIC Column		C18 Column	
		ESI^+^	ESI^−^	ESI^+^	ESI^−^	ESI^+^	ESI^−^	ESI^+^	ESI^−^
Ag	Positive	4	-	1	1	-	-	-	-
	Negative	3	8	14	13	-	-	-	-
As	Positive	147	1648	3855	2606	312	2367	6540	4521
	Negative	90	172	193	47	174	266	435	109
Ba	Positive	2	-	2	-	-	-	-	-
	Negative	-	-	-	-	-	-	-	1
Ca	Positive	25	396	1363	783	136	459	1964	1039
	Negative	10	28	57	25	22	38	106	31
Cd	Positive	-	4	96	14	3	49	765	184
	Negative	1	-	1	2	17	19	22	11
Ce	Positive	2	-	4	7	-	-	-	1
	Negative	-	-	1	-	-	-	-	-
Cr	Positive	225	70	333	248	-	-	-	1
	Negative	-	2	5	3	-	-	-	-
Cu	Positive	-	1	31	6	1	15	88	16
	Negative	22	2	90	6	54	14	131	14
Eu	Positive	32	45	68	59	-	-	-	-
	Negative	1	2	11	4	-	-	-	-
Fe	Positive	5	2	4	4	-	-	-	-
	Negative	1	-	-	-	-	-	-	-
Mg	Positive	544	2319	9765	6077	1815	3652	14,488	8934
	Negative	259	270	396	164	359	361	754	299
Nd	Positive	75	115	336	190	-	-	-	-
	Negative	-	3	12	6	-	-	-	-
Ni	Positive	-	-	3	3	-	1	9	7
	Negative	-	-	-	-	-	-	-	6
Pb	Positive	41	306	2381	1205	66	138	1396	783
	Negative	49	26	100	35	64	22	111	28
Rb	Positive	356	1745	5363	3371	1004	3015	8953	5561
	Negative	183	248	299	100	276	367	640	217
Se	Positive	164	1418	5335	3330	612	2418	8967	5117
	Negative	180	228	288	93	282	341	593	231
Sr	Positive	161	1281	5149	3200	656	2033	7833	4583
	Negative	88	130	176	67	147	177	346	116
Tb	Positive	-	-	-	-	-	-	-	-
	Negative	-	-	3	-	-	1	1	-
Tl	Positive	5	23	32	13	80	130	67	26
	Negative	-	3	1	-	12	17	4	2
Y	Positive	71	121	329	191	-	-	-	-
	Negative	-	1	3	1	-	-	-	-
Zn	Positive	2	-	-	-	-	-	3	1
	Negative	-	-	-	-	-	-	-	-

## Data Availability

The original data presented in the study are openly available from this link: https://drive.google.com/drive/folders/1MuCMbQDrJUcD2Kxj9-az6VcMR4CFJmC0 (Access date: 29 May 2025).

## Supplementary Materials

The following supporting information can be downloaded at: https://www.mdpi.com/article/10.3390/metabo15070456/s1, Figure S1: Sunburst plots of chemo-ontology profiles; Figure S2: Annotated metabolite features linearly associated with urinary metal element; Figure S3: Annotated metabolite features nonlinearly associated (4PL model) with urinary metal element; Table S1: Summary of MS1 feature detection and MS2 based annotation.

## Abbreviations

The following abbreviations are used in this manuscript:

E-waste	Electronic waste
LC	Liquid chromatography
UHPLC	Ultra-high-performance liquid chromatography
HILIC	Hydrophilic interaction liquid chromatography
MS	Mass spectrometry
MS2	Tandem mass spectrometry
HRMS	High-resolution mass spectrometry
ICP-MS	Inductively coupled plasma mass spectrometer
ESI	Electrospray ionization
4PL	Four-parameter logistic
BMI	Body mass index
EC50	Half maximal effective concentration
BMD	Benchmark dose
BMDL	Benchmark dose lower confidence limit
Ag	Silver
As	Arsenic
Ba	Barium
Ca	Calcium
Cd	Cadmium
Ce	Cerium
Cr	Chromium
Cu	Copper
Eu	Europium
Fe	Iron
La	Lanthanum
Mg	Magnesium
Nd	Neodymium
Ni	Nickel
Pb	Lead
Rb	Rubidium
Se	Selenium
Sr	Strontium
Tb	Terbium
Tl	Thallium
Y	Yttrium
Zn	Zinc
CoA	Coenzyme A
