# Supplementary material for: Integrative Modeling of Urinary Metabolomics and Metal Exposure Reveals Systemic Impacts of Electronic Waste in Exposed Populations
Source: Metabolites. 2025 Jul 5;15(7):456. doi: 10.3390/metabo15070456 (PMC12300153; doi:10.3390/metabo15070456)
Supplement: Supplementary file 1 [file metabolites-15-00456-s001.zip › metabolites-3701688-SI.pdf]

metabolites

MDPI

Supplementary Materials

Integrative Modeling of Urinary Metabolomics and Metal Exposure Reveals Systemic Impacts of Electronic Waste in Exposed Populations

Fiona Hui [1], Zhiqiang Pang [1], Charles Viau [1], Gerd U. Balcke [2], Julius N. Fobil [3,4], Niladri Basu [1] and Jianguo Xia [1,5,*]

[1] Faculty of Agricultural and Environmental Sciences, McGill University, Ste-Anne-de-Bellevue, QC, Canada; fiona.hui@mail.mcgill.ca (F.H.); charles.viau@mcgill.ca (C.V.); niladri.basu@mcgill.ca (N.B.)
[2] Metacom Program Center, Leibniz Institute of Plant Biochemistry, Halle, Germany; gerd.balcke@ipb-halle.de
[3] School of Public Health, University of Ghana, Legon Accra, Ghana; jfobil@ug.edu.gh
[4] West Africa Center for Global Environmental & Occupational Health, College of Health Sciences, Legon, Accra, Ghana
[5] Department of Microbiology and Immunology, McGill University, Montreal, QC, Canada
* Correspondence: jeff.xia@mcgill.ca; Tel.: +1-514-398-8668

Table S1. MS1 feature detection and MS2 based annotation.

MS1: *asari* preferred features

		Negative Mode	Positive Mode
E-waste	HILIC	47,508	54,865
	C18	110,828	136,768
Control	HILIC	29,706	38,762
	C18	76,914	99,144

MS2: spectral library matching

		Negative Mode	Positive Mode
E-waste	HILIC	1,644	1,420
	C18	2,555	2,055
Control	HILIC	1,246	1,145
	C18	2,216	2,165

MS2: chemical ontology prediction

		Negative Mode	Positive Mode
E-waste	HILIC	1,240	1,040
	C18	2,388	1,939
Control	HILIC	319	306
	C18	753	503

Control Group

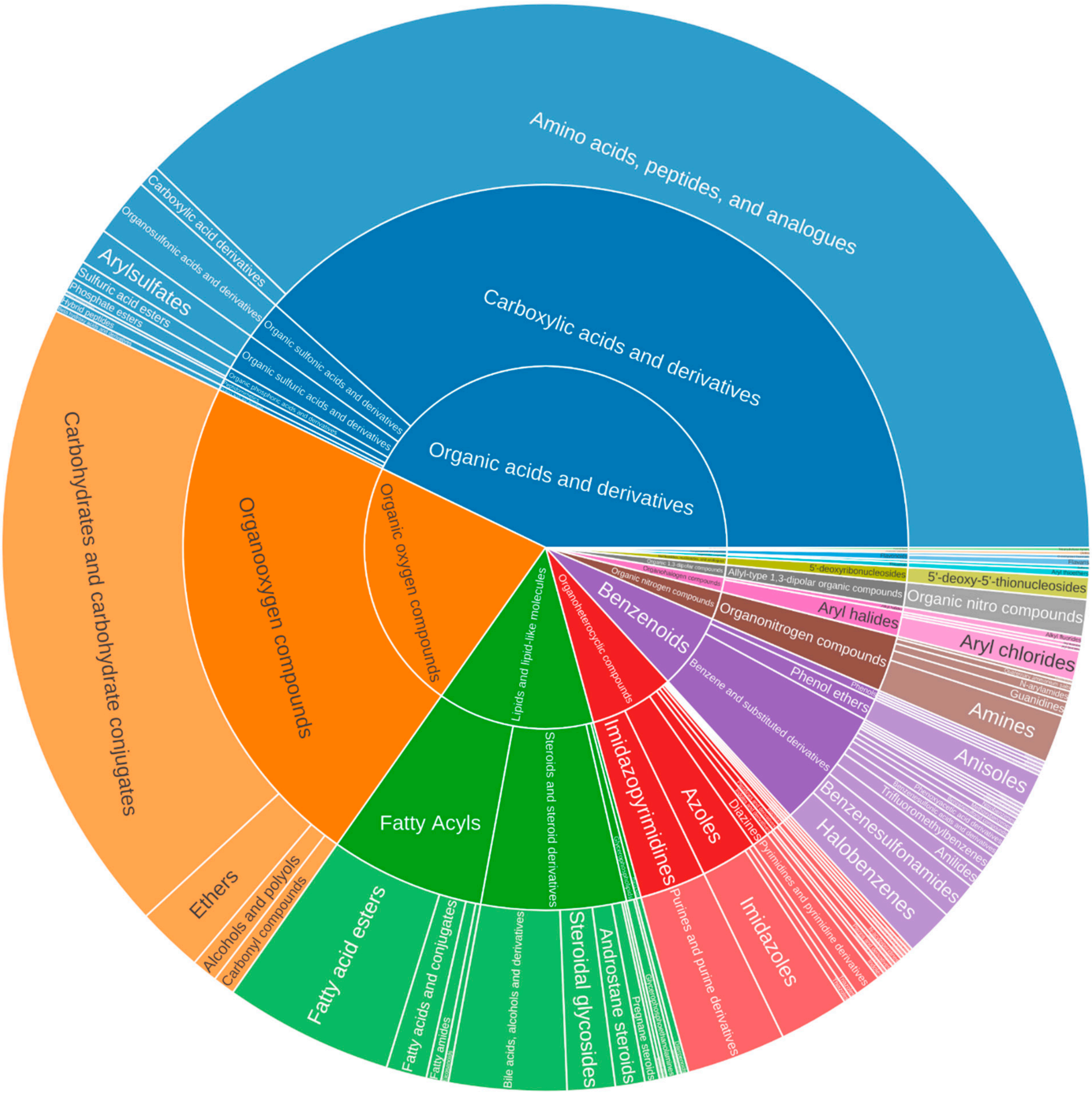

E-waste workers

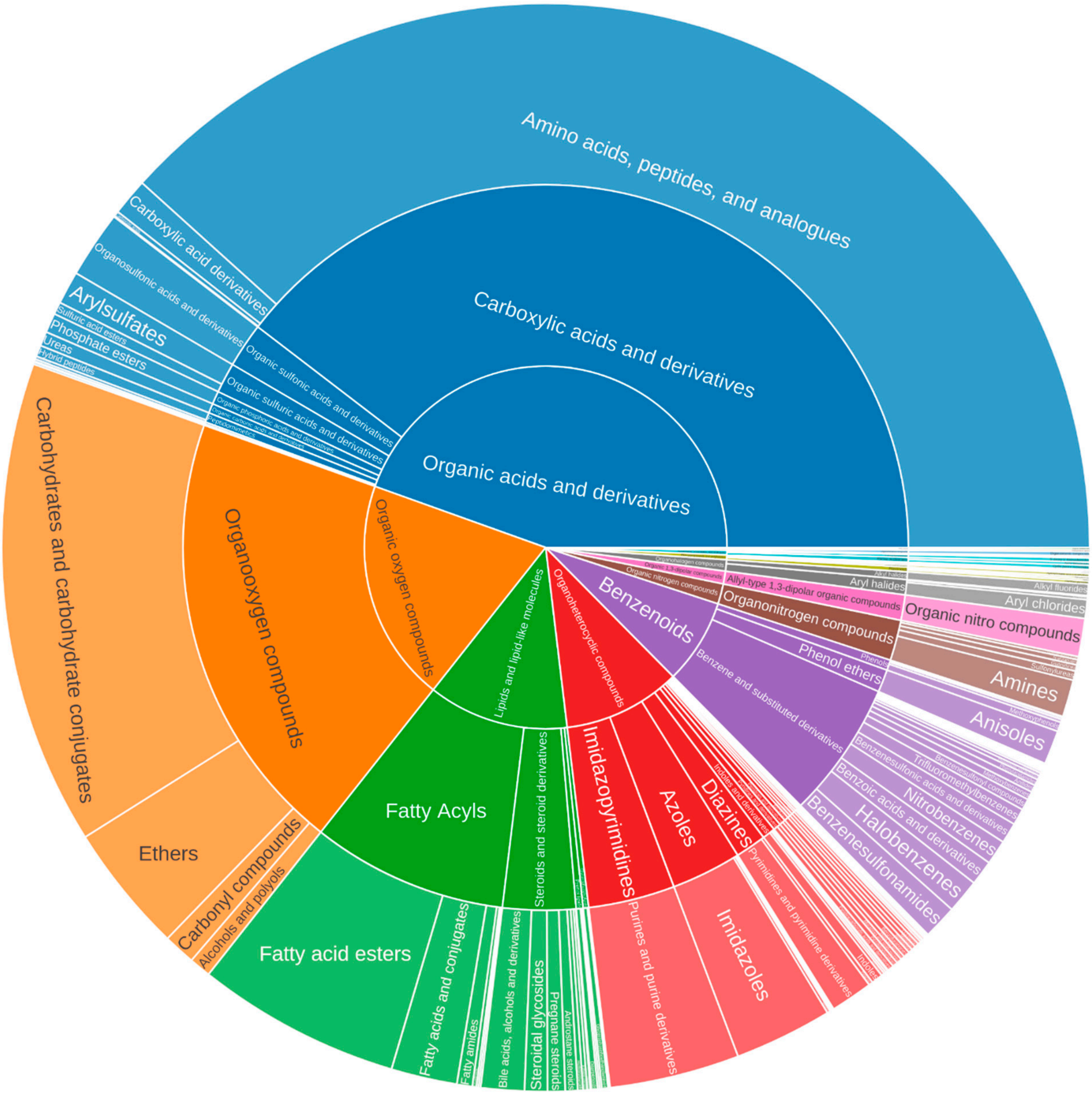

Figure S1. Sunburst plots of chemo-ontology profiles.

As

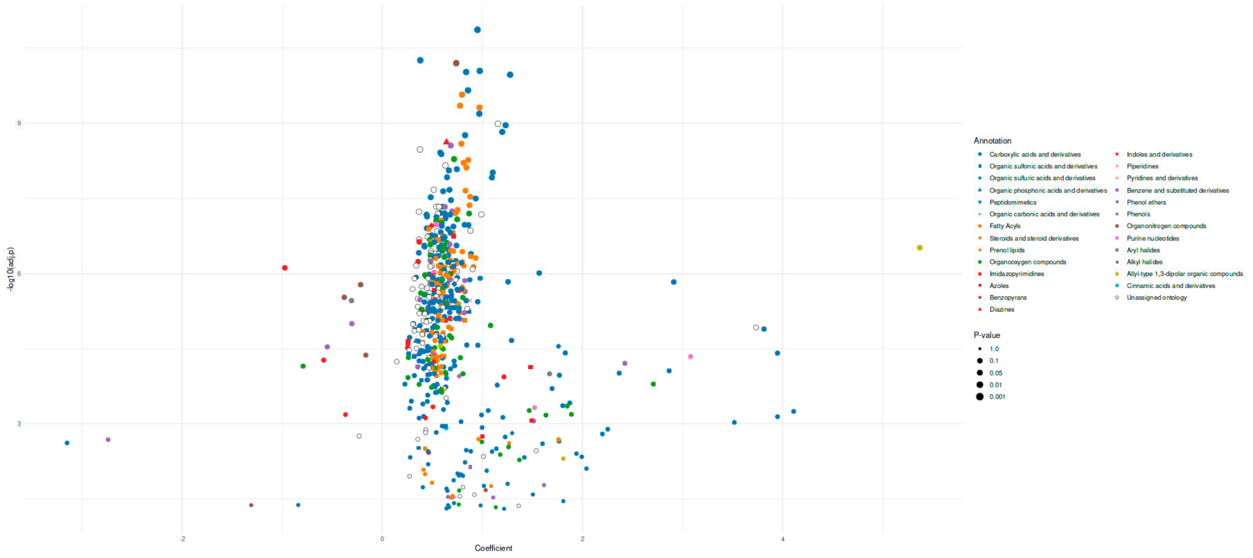
Annotation
Carboxylic acids and derivatives
Organic sulfonic acids and derivatives
Organic sulfuric acids and derivatives
Organic phosphoric acids and derivatives
Peptidomimetics
Organic carbonic acids and derivatives
Fatty Acyls
Steroids and steroid derivatives
Prenol lipids
Organooxygen compounds
Imidazopyrimidines
Azoles
Benzopyrans
Diazines
Indoles and derivatives
Piperidines
Pyridines and derivatives
Benzene and substituted derivatives
Phenol ethers
Phenols
Organonitrogen compounds
Purine nucleotides
Aryl halides
Alkyl halides
Allyl-type 1,3-dipolar organic compounds
Cinnamic acids and derivatives
Unassigned ontology
P-value
1.0
0.1
0.05
0.01
0.001
-log10(adj.p)
Coefficient

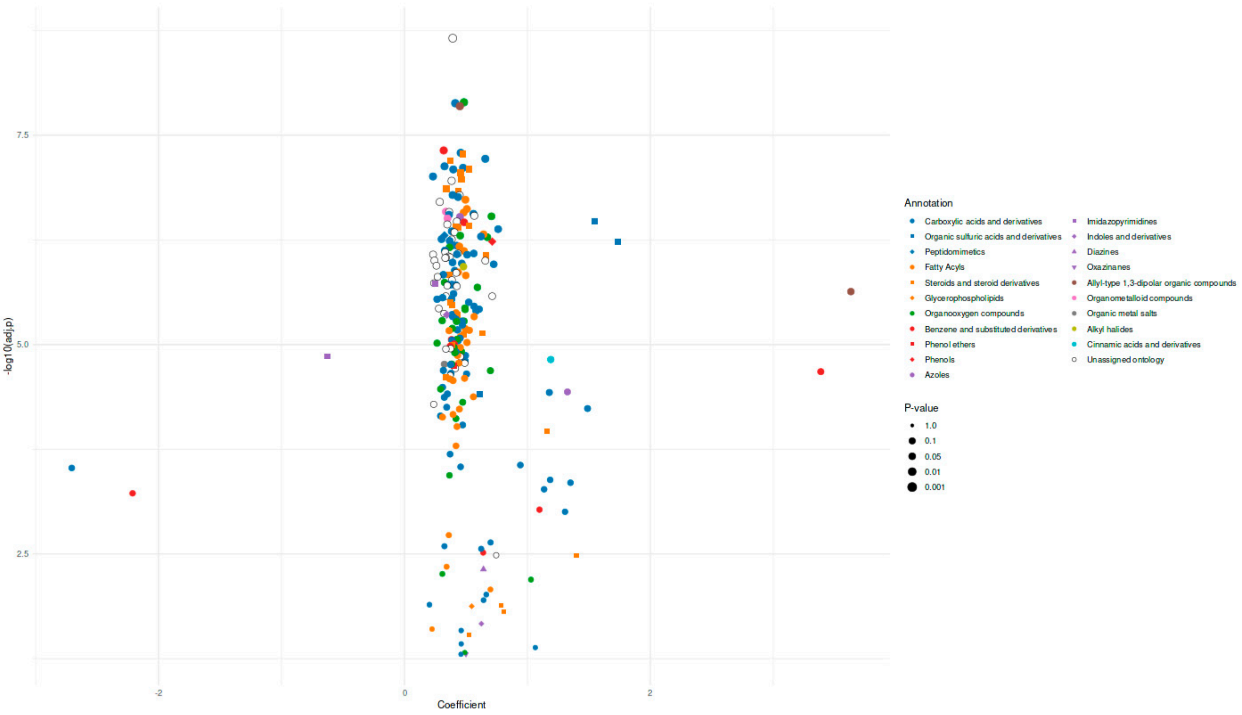
Annotation
Carboxylic acids and derivatives
Organic sulfuric acids and derivatives
Peptidomimetics
Fatty Acyls
Steroids and steroid derivatives
Glycerophospholipids
Organooxygen compounds
Benzene and substituted derivatives
Phenol ethers
Phenols
Azoles
Imidazopyrimidines
Indoles and derivatives
Diazines
Oxazinanes
Allyl-type 1,3-dipolar organic compounds
Organometalloid compounds
Organic metal salts
Alkyl halides
Cinnamic acids and derivatives
Unassigned ontology
P-value
1.0
0.1
0.05
0.01
0.001
-log10(adj.p)
Coefficient

Ca

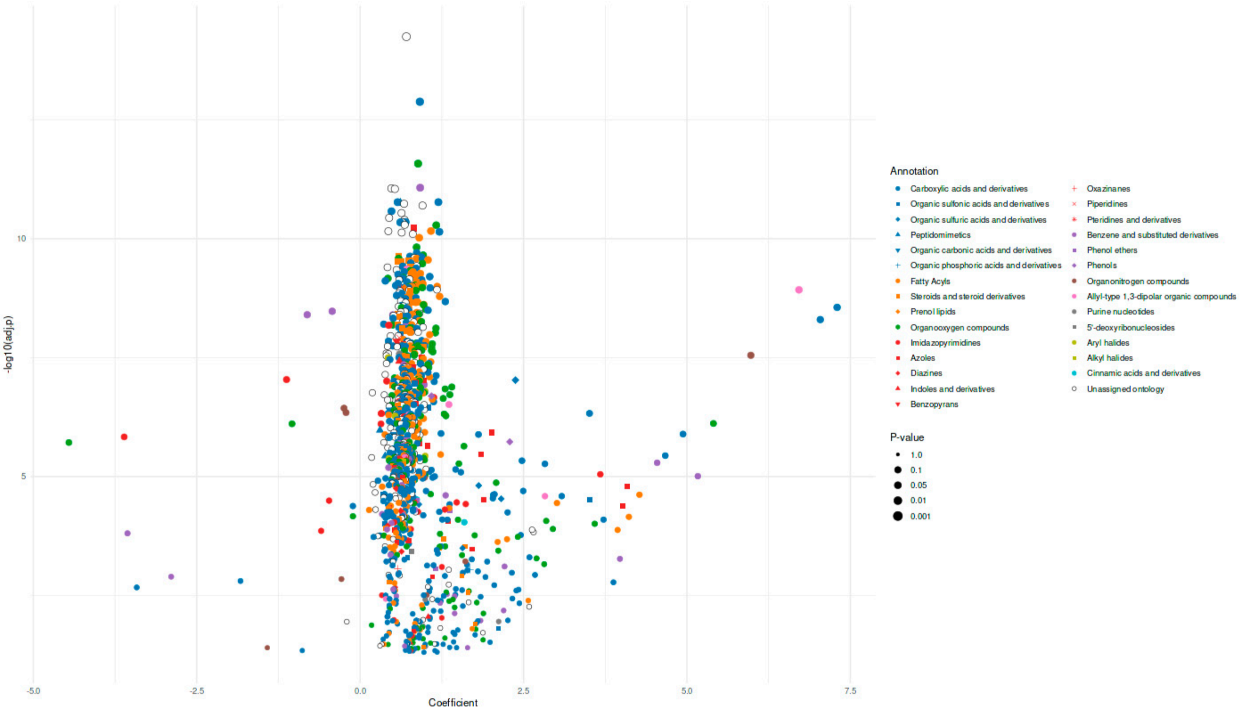
-log10(adj.p)
10
5
-5.0
-2.5
0.0
2.5
5.0
7.5
Coefficient
Annotation
Carboxylic acids and derivatives
Organic sulfonic acids and derivatives
Organic sulfuric acids and derivatives
Peptidomimetics
Organic carbonic acids and derivatives
Organic phosphoric acids and derivatives
Fatty Acyls
Steroids and steroid derivatives
Prenol lipids
Organooxygen compounds
Imidazopyrimidines
Azoles
Diazines
Indoles and derivatives
Benzopyrans
Oxazinanes
Piperidines
Pteridines and derivatives
Benzene and substituted derivatives
Phenol ethers
Phenols
Organonitrogen compounds
Allyl-type 1,3-dipolar organic compounds
Purine nucleotides
5'-deoxyribonucleosides
Aryl halides
Alkyl halides
Cinnamic acids and derivatives
Unassigned ontology
P-value
1.0
0.1
0.05
0.01
0.001

Mg

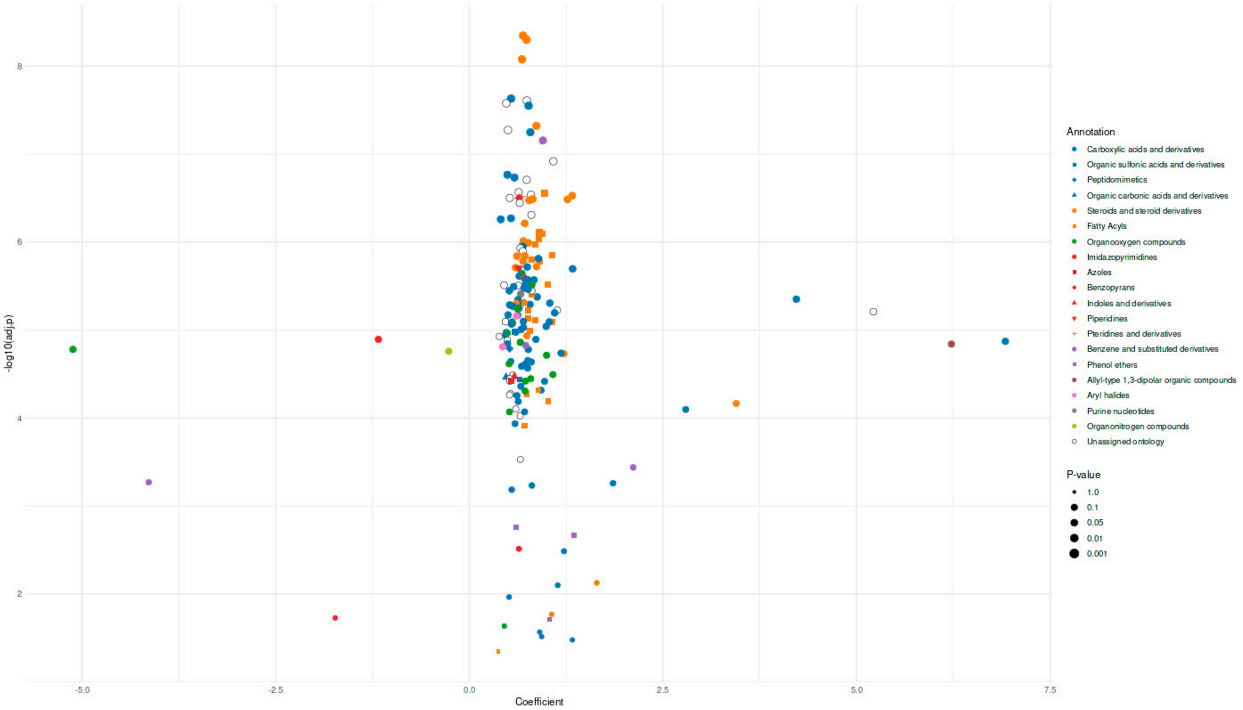
-log10(adj.p)
8
6
4
2
-5.0
-2.5
0.0
2.5
5.0
7.5
Coefficient
Annotation
Carboxylic acids and derivatives
Organic sulfonic acids and derivatives
Peptidomimetics
Organic carbonic acids and derivatives
Steroids and steroid derivatives
Fatty Acyls
Organooxygen compounds
Imidazopyrimidines
Azoles
Benzopyrans
Indoles and derivatives
Piperidines
Pteridines and derivatives
Benzene and substituted derivatives
Phenol ethers
Allyl-type 1,3-dipolar organic compounds
Aryl halides
Purine nucleotides
Organonitrogen compounds
Unassigned ontology
P-value
1.0
0.1
0.05
0.01
0.001

Pb

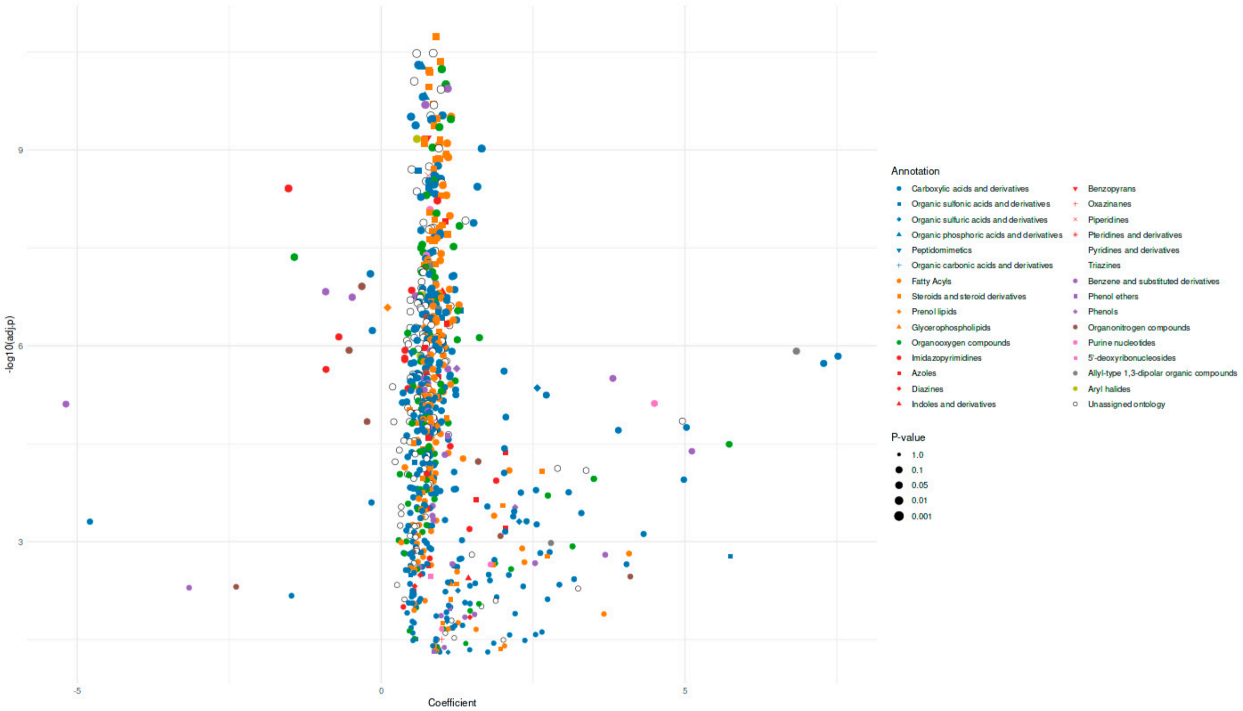
-log10(adj.p)
Coefficient
Annotation
Carboxylic acids and derivatives
Organic sulfonic acids and derivatives
Organic sulfuric acids and derivatives
Organic phosphoric acids and derivatives
Peptidomimetics
Organic carbonic acids and derivatives
Fatty Acyls
Steroids and steroid derivatives
Prenol lipids
Glycerophospholipids
Organooxygen compounds
Imidazopyrimidines
Azoles
Diazines
Indoles and derivatives
Benzopyrans
Oxazinanes
Piperidines
Pteridines and derivatives
Pyridines and derivatives
Triazines
Benzene and substituted derivatives
Phenol ethers
Phenols
Organonitrogen compounds
Purine nucleotides
5'-deoxyribonucleosides
Allyl-type 1,3-dipolar organic compounds
Aryl halides
Unassigned ontology
P-value
1.0
0.1
0.05
0.01
0.001
Rb

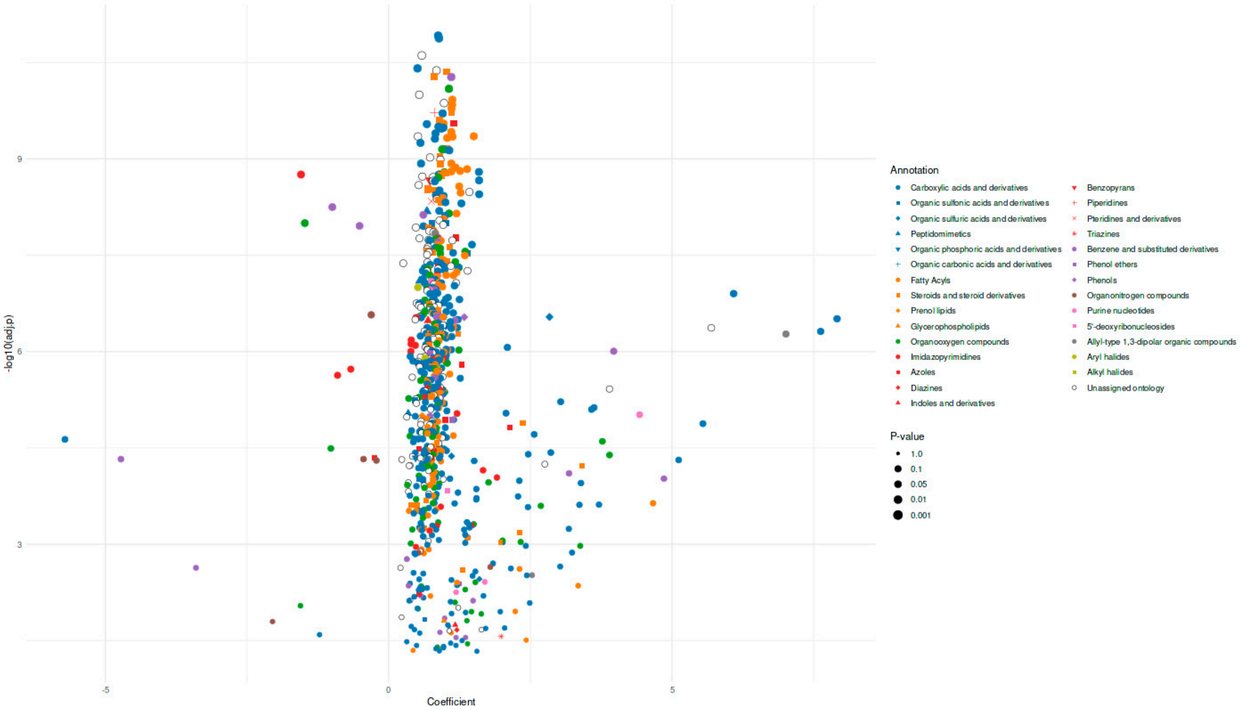
-log10(adj.p)
Coefficient
Annotation
Carboxylic acids and derivatives
Organic sulfonic acids and derivatives
Organic sulfuric acids and derivatives
Peptidomimetics
Organic phosphoric acids and derivatives
Organic carbonic acids and derivatives
Fatty Acyls
Steroids and steroid derivatives
Prenol lipids
Glycerophospholipids
Organooxygen compounds
Imidazopyrimidines
Azoles
Diazines
Indoles and derivatives
Benzopyrans
Piperidines
Pteridines and derivatives
Triazines
Benzene and substituted derivatives
Phenol ethers
Phenols
Organonitrogen compounds
Purine nucleotides
5'-deoxyribonucleosides
Allyl-type 1,3-dipolar organic compounds
Aryl halides
Alkyl halides
Unassigned ontology
P-value
1.0
0.1
0.05
0.01
0.001
Se

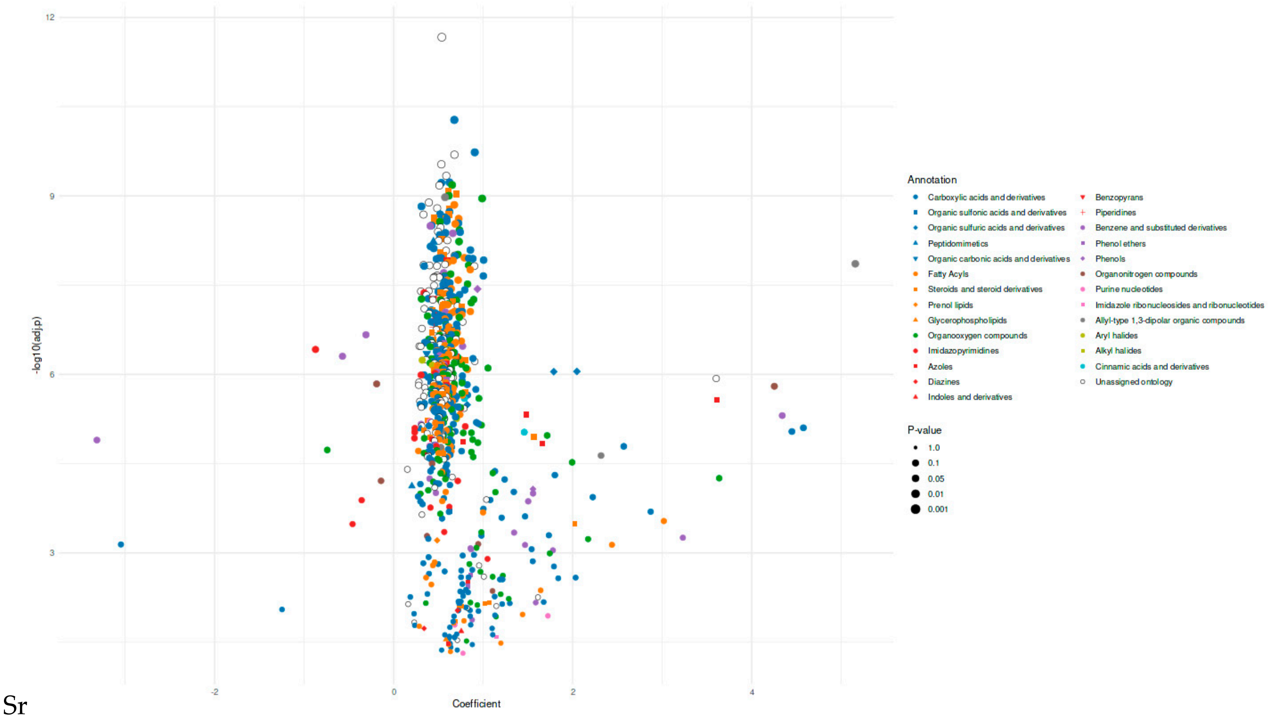

Sr

Figure S2. Annotated metabolite features linearly associated with urinary metal element.

As

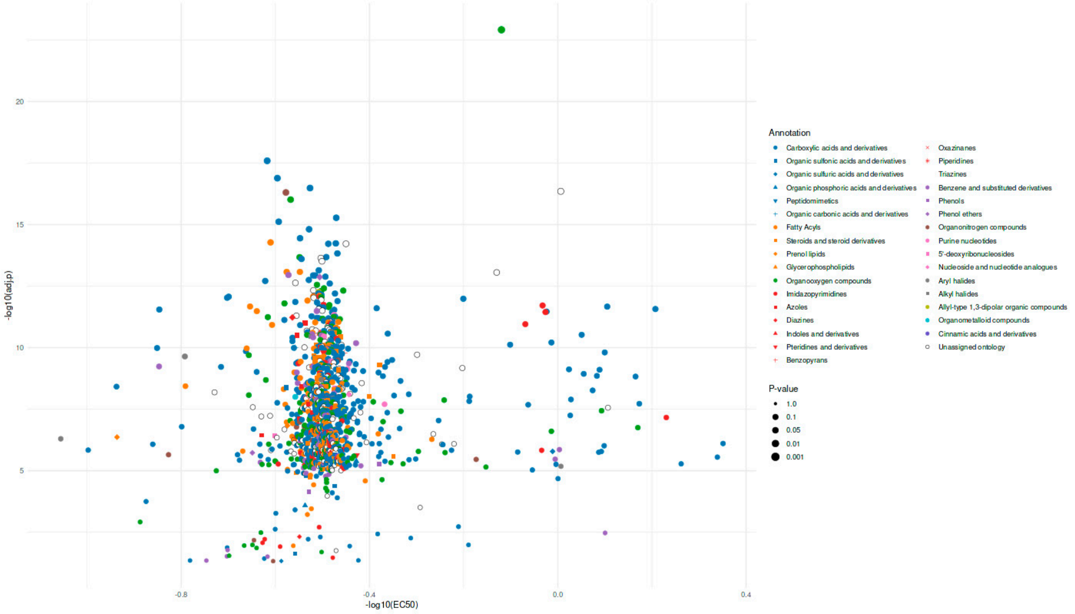
-log10(adj.p)
20
15
10
5
-0.8
-0.4
0.0
0.4
-log10(EC50)
Annotation
Carboxylic acids and derivatives
Organic sulfonic acids and derivatives
Organic sulfuric acids and derivatives
Organic phosphoric acids and derivatives
Peptidomimetics
Organic carbonic acids and derivatives
Fatty Acyls
Steroids and steroid derivatives
Prenol lipids
Glycerophospholipids
Organooxygen compounds
Imidazopyrimidines
Azoles
Diazines
Indoles and derivatives
Pteridines and derivatives
Benzopyrans
Oxazinanes
Piperidines
Triazines
Benzene and substituted derivatives
Phenols
Phenol ethers
Organonitrogen compounds
Purine nucleotides
5'-deoxyribonucleosides
Nucleoside and nucleotide analogues
Aryl halides
Alkyl halides
Allyl-type 1,3-dipolar organic compounds
Organometalloid compounds
Cinnamic acids and derivatives
Unassigned ontology
P-value
1.0
0.1
0.05
0.01
0.001

Ca

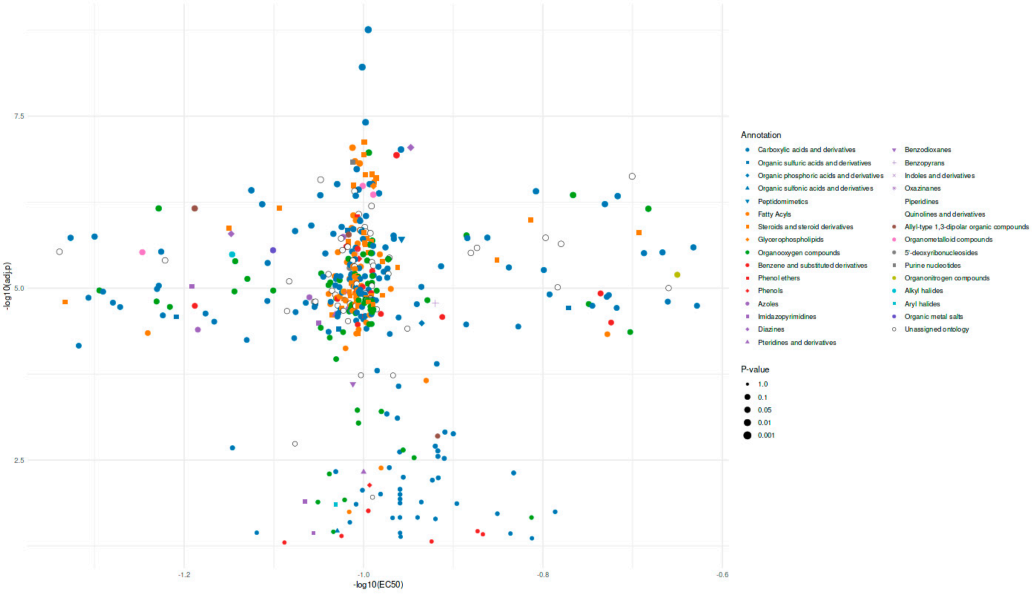
-log10(adj.p)
7.5
5.0
2.5
-1.2
-1.0
-0.8
-0.6
-log10(EC50)
Annotation
Carboxylic acids and derivatives
Organic sulfuric acids and derivatives
Organic phosphoric acids and derivatives
Organic sulfonic acids and derivatives
Peptidomimetics
Fatty Acyls
Steroids and steroid derivatives
Glycerophospholipids
Organooxygen compounds
Benzene and substituted derivatives
Phenol ethers
Phenols
Azoles
Imidazopyrimidines
Diazines
Pteridines and derivatives
Benzodioxanes
Benzopyrans
Indoles and derivatives
Oxazinanes
Piperidines
Quinolines and derivatives
Allyl-type 1,3-dipolar organic compounds
Organometalloid compounds
5'-deoxyribonucleosides
Purine nucleotides
Organonitrogen compounds
Alkyl halides
Aryl halides
Organic metal salts
Unassigned ontology
P-value
1.0
0.1
0.05
0.01
0.001

Cd

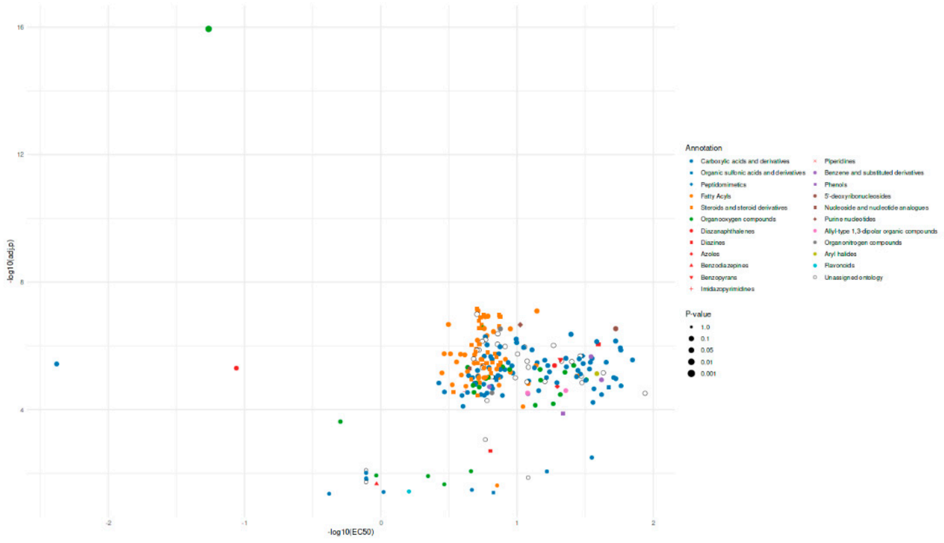
16
12
8
4
-log10(adj.p)
-2
-1
0
1
2
-log10(EC50)
Annotation
Carboxylic acids and derivatives
Organic sulfonic acids and derivatives
Peptidomimetics
Fatty Acyls
Steroids and steroid derivatives
Organooxygen compounds
Diazanaphthalenes
Diazines
Azoles
Benzodiazepines
Benzopyrans
Imidazopyrimidines
Piperidines
Benzene and substituted derivatives
Phenols
5'-deoxyribonucleosides
Nucleoside and nucleotide analogues
Purine nucleotides
Allyl-type 1,3-dipolar organic compounds
Organonitrogen compounds
Aryl halides
Flavonoids
Unassigned ontology
P-value
1.0
0.1
0.05
0.01
0.001

Cu

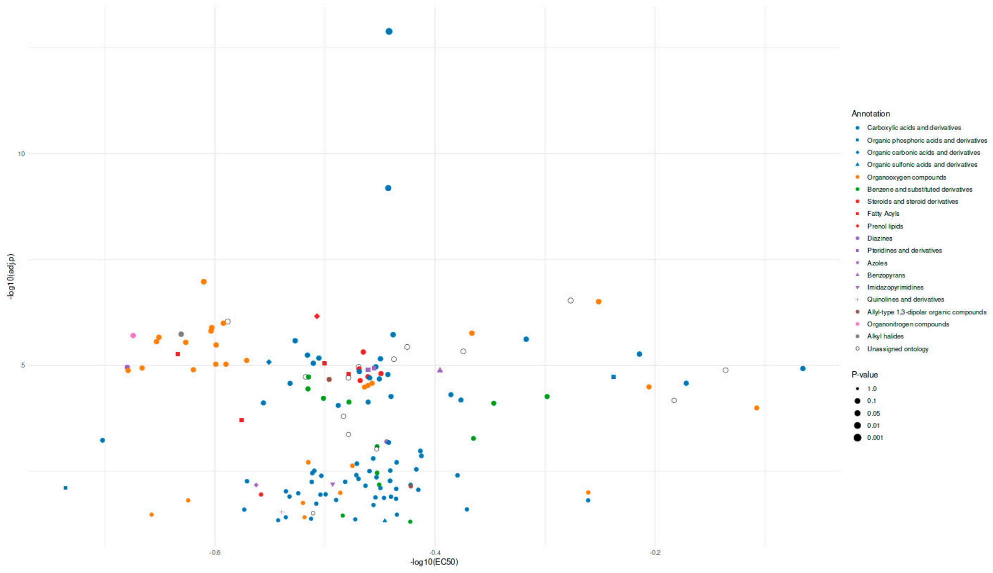
10
5
-log10(adj.p)
-0.6
-0.4
-0.2
-log10(EC50)
Annotation
Carboxylic acids and derivatives
Organic phosphoric acids and derivatives
Organic carbonic acids and derivatives
Organic sulfonic acids and derivatives
Organooxygen compounds
Benzene and substituted derivatives
Steroids and steroid derivatives
Fatty Acyls
Prenol lipids
Diazines
Pteridines and derivatives
Azoles
Benzopyrans
Imidazopyrimidines
Quinolines and derivatives
Allyl-type 1,3-dipolar organic compounds
Organonitrogen compounds
Alkyl halides
Unassigned ontology
P-value
1.0
0.1
0.05
0.01
0.001

Mg

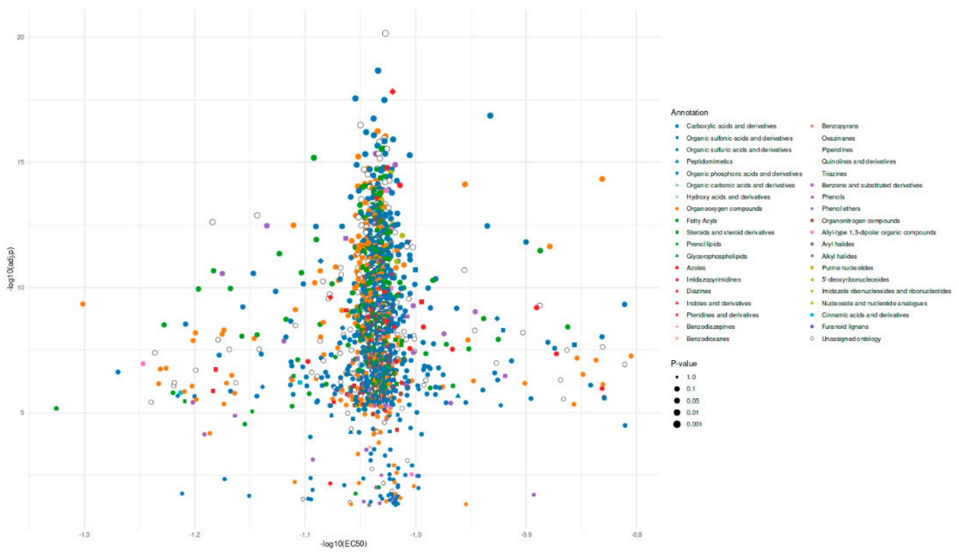
20
15
10
5
-log10(adj.p)
-1.3
-1.2
-1.1
-1.0
-0.9
-0.8
-log10(EC50)
Annotation
Carboxylic acids and derivatives
Organic sulfonic acids and derivatives
Organic sulfuric acids and derivatives
Peptidomimetics
Organic phosphoric acids and derivatives
Organic carbonic acids and derivatives
Hydroxy acids and derivatives
Organooxygen compounds
Fatty Acyls
Steroids and steroid derivatives
Prenol lipids
Glycerophospholipids
Azoles
Imidazopyrimidines
Diazines
Indoles and derivatives
Pteridines and derivatives
Benzodiazepines
Benzodioxanes
Benzopyrans
Oxazinanes
Piperidines
Quinolines and derivatives
Triazines
Benzene and substituted derivatives
Phenols
Phenol ethers
Organonitrogen compounds
Allyl-type 1,3-dipolar organic compounds
Aryl halides
Alkyl halides
Purine nucleotides
5'-deoxyribonucleosides
Imidazole ribonucleosides and ribonucleotides
Nucleoside and nucleotide analogues
Cinnamic acids and derivatives
Furanoid lignans
Unassigned ontology
P-value
1.0
0.1
0.05
0.01
0.001

Pb

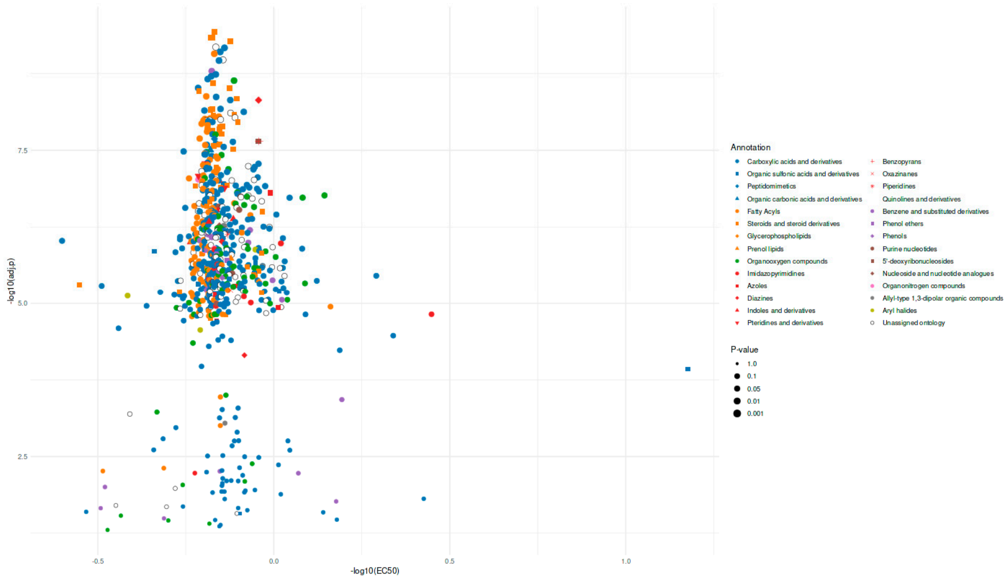
7.5
5.0
2.5
-log10(adj.p)
-0.5
0.0
0.5
1.0
-log10(EC50)
Annotation
Carboxylic acids and derivatives
Organic sulfonic acids and derivatives
Peptidomimetics
Organic carbonic acids and derivatives
Fatty Acyls
Steroids and steroid derivatives
Glycerophospholipids
Prenol lipids
Organooxygen compounds
Imidazopyrimidines
Azoles
Diazines
Indoles and derivatives
Pteridines and derivatives
Benzopyrans
Oxazinanes
Piperidines
Quinolines and derivatives
Benzene and substituted derivatives
Phenol ethers
Phenols
Purine nucleotides
5'-deoxyribonucleosides
Nucleoside and nucleotide analogues
Organonitrogen compounds
Allyl-type 1,3-dipolar organic compounds
Aryl halides
Unassigned ontology
P-value
1.0
0.1
0.05
0.01
0.001

Rb

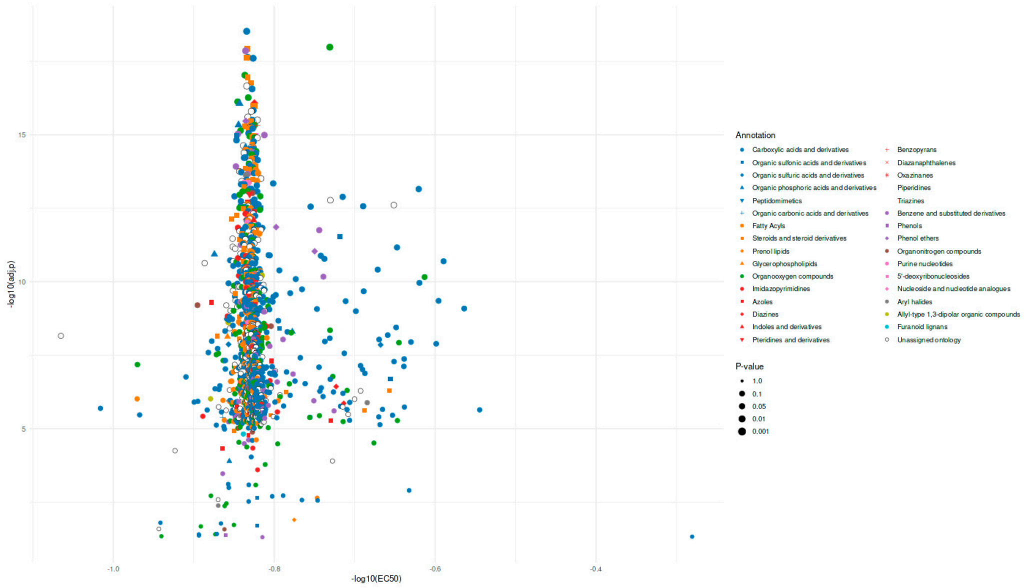
15
10
5
-log10(adj.p)
-1.0
-0.8
-0.6
-0.4
-log10(EC50)
Annotation
Carboxylic acids and derivatives
Organic sulfonic acids and derivatives
Organic sulfuric acids and derivatives
Organic phosphoric acids and derivatives
Peptidomimetics
Organic carbonic acids and derivatives
Fatty Acyls
Steroids and steroid derivatives
Prenol lipids
Glycerophospholipids
Organooxygen compounds
Imidazopyrimidines
Azoles
Diazines
Indoles and derivatives
Pteridines and derivatives
Benzopyrans
Diazanaphthalenes
Oxazinanes
Piperidines
Triazines
Benzene and substituted derivatives
Phenols
Phenol ethers
Organonitrogen compounds
Purine nucleotides
5'-deoxyribonucleosides
Nucleoside and nucleotide analogues
Aryl halides
Allyl-type 1,3-dipolar organic compounds
Furanoid lignans
Unassigned ontology
P-value
1.0
0.1
0.05
0.01
0.001

Se

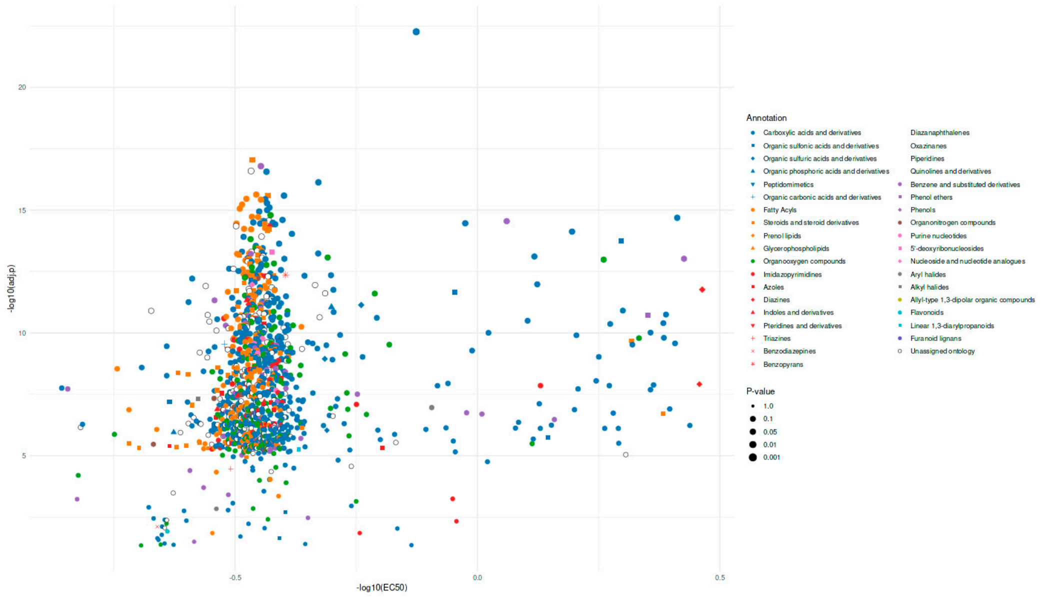
20
15
10
5
-log10(adj.p)
-0.5
0.0
0.5
-log10(EC50)
Annotation
Carboxylic acids and derivatives
Organic sulfonic acids and derivatives
Organic sulfuric acids and derivatives
Organic phosphoric acids and derivatives
Peptidomimetics
Organic carbonic acids and derivatives
Fatty Acyls
Steroids and steroid derivatives
Prenol lipids
Glycerophospholipids
Organooxygen compounds
Imidazopyrimidines
Azoles
Diazines
Indoles and derivatives
Pteridines and derivatives
Triazines
Benzodiazepines
Benzopyrans
Diazanaphthalenes
Oxazinanes
Piperidines
Quinolines and derivatives
Benzene and substituted derivatives
Phenol ethers
Phenols
Organonitrogen compounds
Purine nucleotides
5'-deoxyribonucleosides
Nucleoside and nucleotide analogues
Aryl halides
Alkyl halides
Allyl-type 1,3-dipolar organic compounds
Flavonoids
Linear 1,3-diarylpropanoids
Furanoid lignans
Unassigned ontology
P-value
1.0
0.1
0.05
0.01
0.001

Sr

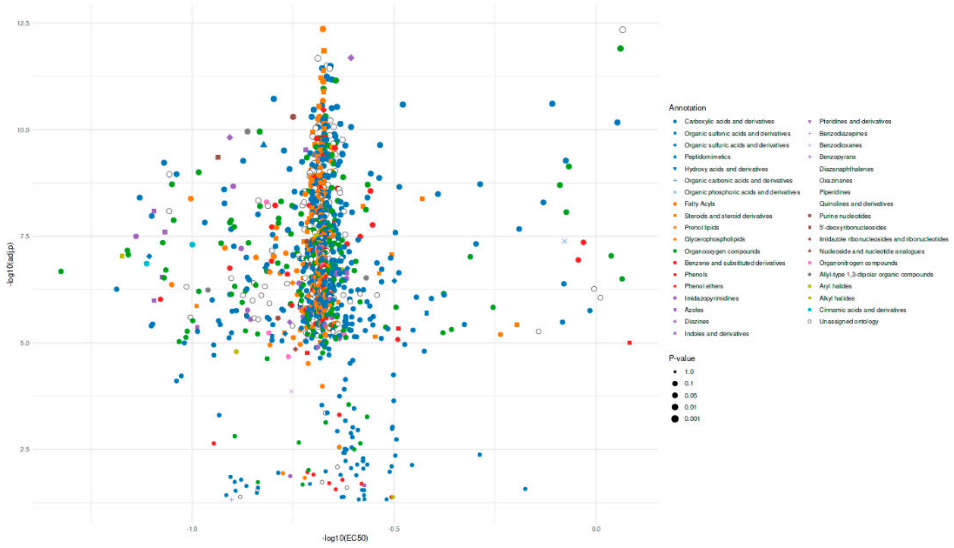

Zn

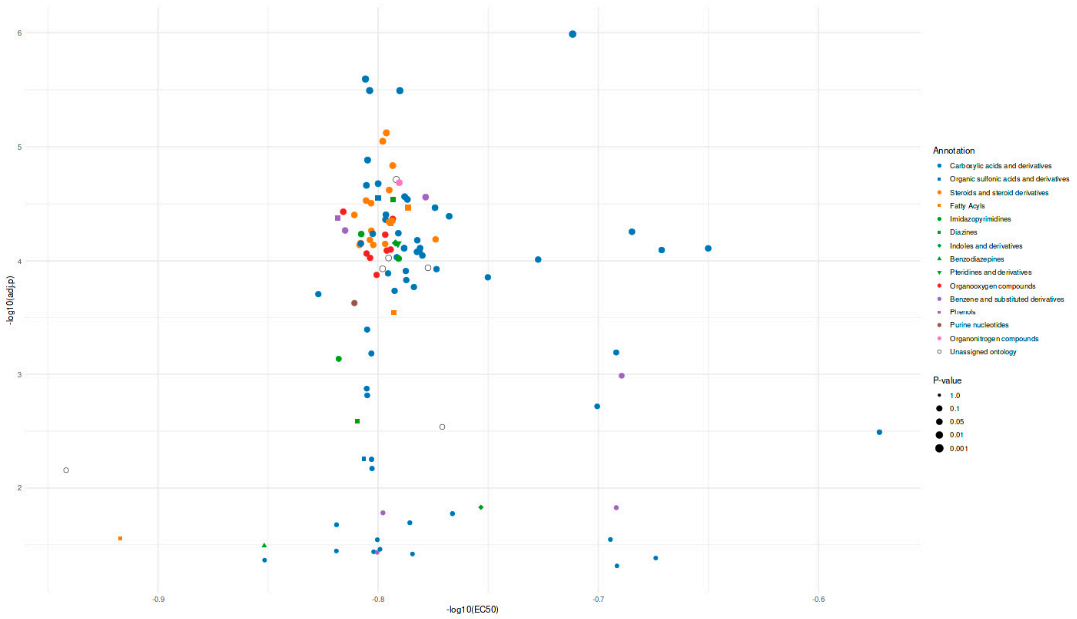

Figure S3. Annotated metabolite features non-linearly associated (4PL model) with urinary metal element.
